# *Schistosoma mansoni* Egg, Adult Male and Female Comparative Gene Expression Analysis and Identification of Novel Genes by RNA-Seq

**DOI:** 10.1371/journal.pntd.0004334

**Published:** 2015-12-31

**Authors:** Letícia Anderson, Murilo S. Amaral, Felipe Beckedorff, Lucas F. Silva, Bianca Dazzani, Katia C. Oliveira, Giulliana T. Almeida, Monete R. Gomes, David S. Pires, João C. Setubal, Ricardo DeMarco, Sergio Verjovski-Almeida

**Affiliations:** 1 Departamento de Bioquímica, Instituto de Química, Universidade de São Paulo, São Paulo, Brazil; 2 Instituto Butantan, São Paulo, SP, Brazil; 3 Núcleo de Enteroparasitas, Centro de Parasitologia e Micologia, Instituto Adolfo Lutz, São Paulo, SP, Brazil; 4 Virginia Bioinformatics Institute, Virginia Polytechnic Institute and State University, Blacksburg, Virginia, United States of America; 5 Instituto de Física de São Carlos, Universidade de São Paulo, São Carlos, SP, Brazil; University of Melbourne, AUSTRALIA

## Abstract

**Background:**

Schistosomiasis is one of the most prevalent parasitic diseases worldwide and is a public health problem. *Schistosoma mansoni* is the most widespread species responsible for schistosomiasis in the Americas, Middle East and Africa. Adult female worms (mated to males) release eggs in the hepatic portal vasculature and are the principal cause of morbidity. Comparative separate transcriptomes of female and male adult worms were previously assessed with using microarrays and Serial Analysis of Gene Expression (SAGE), thus limiting the possibility of finding novel genes. Moreover, the egg transcriptome was analyzed only once with limited bacterially cloned cDNA libraries.

**Methodology/Principal findings:**

To compare the gene expression of *S*. *mansoni* eggs, females, and males, we performed RNA-Seq on these three parasite forms using 454/Roche technology and reconstructed the transcriptome using Trinity *de novo* assembly. The resulting contigs were mapped to the genome and were cross-referenced with predicted Smp genes and H3K4me3 ChIP-Seq public data. For the first time, we obtained separate, unbiased gene expression profiles for *S*. *mansoni* eggs and female and male adult worms, identifying enriched biological processes and specific enriched functions for each of the three parasite forms. Transcripts with no match to predicted genes were analyzed for their protein-coding potential and the presence of an encoded conserved protein domain. A set of 232 novel protein-coding genes with putative functions related to reproduction, metabolism, and cell biogenesis was detected, which contributes to the understanding of parasite biology.

**Conclusions/Significance:**

Large-scale RNA-Seq analysis using *de novo* assembly associated with genome-wide information for histone marks in the vicinity of gene models constitutes a new approach to transcriptome analysis that has not yet been explored in schistosomes. Importantly, all data have been consolidated into a UCSC Genome Browser search- and download-tool (http://schistosoma.usp.br/). This database provides new ways to explore the schistosome genome and transcriptome and will facilitate molecular research on this important parasite.

## Author Summary

Schistosomiasis is a public health problem caused by parasites of the genus *Schistosoma*, of which *S*. *mansoni* is the primary causative agent. The parasite has a complex life cycle; their sexual reproductive stage is dependent on female and male adult worms mating inside the mesenteric circulation of the human host, with the female releasing hundreds of eggs daily. This phase of the life cycle is responsible for the development of pathology, which is proportional to the number of eggs accumulating in the liver and intestine of the human host. Genome and transcriptome sequencing of this parasite represent important advances in schistosome research, but there is still a need for integrated analyses to better understand the biology of this parasite. In this study, we describe the first large-scale transcriptomes of eggs, and female and male adult worms, the parasite forms that are mainly responsible for the pathology of schistosomiasis. We were able to cross-reference the gene transcription regions with promoter regions, thus improving the gene annotations. Moreover, we identified the expression of novel protein-coding genes not yet described in the current genome annotation, advancing the biological knowledge regarding this parasite.

## Introduction

Schistosomiasis is a parasitic disease caused by blood-dwelling worms of the genus *Schistosoma*. It is an important public health problem, with high morbidity and mortality in endemic countries. Over 230 million people worldwide are infected by *Schistosoma* spp., comprising three main species [1]; *Schistosoma mansoni* is the species responsible for infecting people in the Americas, Middle East and Africa [1]. The parasite has a complex life cycle that includes several morphological phenotypes in the intermediate *Biomphalaria* spp. snail host and in the human definitive host, with adult worms having separate sexes, and the mated female worms releasing hundreds of eggs daily in the mesenteric circulation of the human host [2].

In the past decade, genomic tools have helped to reveal relevant molecular players in parasite biology. Thus, the *S*. *mansoni* genome was sequenced [3], followed by a systematically improved high-quality version of the genome [4]; however, the latter still includes over 800 genome sequence fragments and a number of incomplete gene annotations. The first *S*. *mansoni* transcriptome analyses from six different life cycle stages (cercaria, schistosomulum, adult worm, egg, miracidium and germ ball) were performed using first-generation EST bacterial cloning and sequencing technology [5], with limited sequencing depth. Later, with the new second-generation, cloning-independent RNA-Seq techniques, mixed-sex adult worms [4] or male adult worms [6] were studied; however, no separate male and female gene expression was assessed by RNA-Seq.

The comparison of *S*. *mansoni* male and female adult worm transcriptomes was performed only using oligonucleotide microarrays, which use short (60 nt) probes to detect known genes [7], and using Serial Analysis of Gene Expression (SAGE), which sequences short (10–14 bp) SAGE tags [8] that need to be matched to previously known full-length gene sequences to unequivocally assign their identity. In addition, the egg transcriptome was analyzed only once using Sanger sequencing [5], and no additional studies have been performed on this life-cycle stage using unbiased, large-scale sequencing.

In this study we compared egg, female and male transcriptomes of *S*. *mansoni* using large-scale RNA-Seq, which enabled the identification of genes functionally related to these specific developmental forms of the parasite. In addition, we documented the extension of the 5’-ends of hundreds of transcripts over the previous *S*. *mansoni* sequence predictions [4]. We then cross-referenced the new genomic coordinates of these transcript start sites (TSSs) with the genomic coordinates of the publicly available dataset of promoter-associated H3K4me3 histone marks obtained by ChIP-Seq [9], and the coincident coordinates for the TSS and the H3K4me3 provided genome-wide evidence of a possible involvement of this histone modification in the transcriptional regulation of these genes. Moreover, an *in silico* analysis of the *de novo-*assembled transcriptome revealed new protein coding genes with conserved domains not previously predicted in the parasite genome.

## Methods

### Parasite materials

Approximately 200 *S*. *mansoni* (BH strain) adult worm pairs were freshly obtained through the periportal perfusion [10] of hamsters infected 6–8 weeks earlier with 200–300 mixed-sex cercariae. After perfusion, the adult males were separated from the adult females by keeping the parasites in 150 mm culture dishes at room temperature for a period of 15 to 30 min in Advanced RPMI Medium 1640 (Gibco, #12633–012, Thermo Scientific, USA) supplemented with 10% heat-inactivated calf serum (freshly added), 12 mm HEPES (4-(2-hydroxyethyl) piperazine-1-ethanesulfonic acid) pH 7.4, and 1% antibiotic/antimycotic solution (Gibco, #15240–096).

Eggs of *S*. *mansoni* were isolated from the livers of five hamsters, each infected 6–8 weeks earlier with 200–300 cercariae, using the method described by the group of Brindley and collaborators [11]. Immediately before the hamster liver extraction, the *S*. *mansoni* adult worms were collected by periportal perfusion [10].

### PolyA^+^ RNA preparation

PolyA^+^ RNA was extracted from eggs, from 200 adult male worms and from 200 adult female worms using two rounds of the FastTrack MAG Maxi mRNA Isolation Kit (Invitrogen, Thermo Scientific, USA), as described [6], with the following modifications: 1,400 units of RNase Out (Invitrogen) and 20 mM of Vanadyl Ribonucleoside Complexes (VRC) were added to Lysis Buffer L4 at the first step of the sample preparation; at the final binding step, an additional 1,400 units of RNase Out (Invitrogen) was added to the sample while the tube remained in the rotator; treatment with 30 units of DNase I Amplification Grade (Invitrogen) was performed for 45 min at room temperature; and six washings were performed at the final washing step before elution. These modifications resulted in PolyA+ RNA samples with small percentages of rRNA contamination (3 to 7%), as estimated with a Bioanalyzer using the RNA 6000 Pico Kit (Agilent Technologies, Santa Clara, CA, USA).

### Strand-oriented cDNA library preparation

Because the Roche 454 sequencing platform did not provide a protocol for the construction of strand-oriented RNA-Seq libraries, we previously developed a method for generating strand-oriented cDNA libraries for 454 sequencing [6], and here, we have improved the protocol, aiming to correct the tendency toward preferential sampling of the 3′-end of the transcripts verified in the previously described method [6]. In the new strand-oriented 5′-end-first cDNA libraries method, the design of the primers was modified so that sequencing initiates from the 5′-end of the transcripts. The resulting single-stranded cDNA (sscDNA) libraries were PCR amplified (10 cycles of PCR for male and female libraries, and 17 cycles of PCR for egg libraries), and the resulting directional double-stranded cDNA (dscDNA) libraries were purified using AMPure beads (Agencourt, Beckman Coulter, Indianapolis, IN, USA) and quantified using PicoGreen (Invitrogen). A detailed description of the library construction is given in the Supplementary Methods in S1 Text, along with a summary scheme of the procedure (see Fig A in S1 Text).

### Pyrosequencing and data pre-processing

Directional dscDNA libraries generated as described above were sequenced from the 5′-end using the emPCR Titanium Kit and the Titanium Sequencing Kit on a Roche 454 Genome Sequencer FLX instrument, following the manufacturer’s instructions [12]. Data processing used standard 454 software procedures to generate nucleotide sequences and quality scores for all reads. High-quality (minimum Phred score 20) trimmed reads were generated, and the sequence complexity filtering criteria were applied using PRINSEQ [13]. Sequencing data were deposited at the NCBI Sequence Read Archive (SRA) under the accession number SRP063353.

To exclude reads of ribosomal, mitochondrial or transposable element origin, we performed an alignment of reads against rRNA, mitochondrial sequences and 29 *S*. *mansoni* transposon sequences for which there were published curated full-length sequences [14–16] utilizing BLASTn [17], and reads displaying an alignment with an e-value ≤ 10^−15^ were deleted. To evaluate the coverage of the reads, the filtered reads were mapped to the genome with Tophat2 [18], and the distribution along the 5′ to 3′ gene body was assessed using the RSeQC package [19].

### *De novo* transcriptome assembly without a reference genome and the classification of transcripts

The *S*. *mansoni* transcriptome was reconstructed without a reference genome, using all reads from egg, female and male RNA-Seq with a Trinity assembler (Version from April 2014) [20] and a *k-*mer size of 25. This project has been deposited at the Transcriptome Shotgun Assembly (TSA) division of DDBJ/EMBL/GenBank under the accession numbers GDQY00000000 (putative novel genes) and GDUI00000000 (all other contigs). The version described in this paper is the first version, GDQY01000000 and GDUI01000000.

For annotation, the contigs resulting from the Trinity assembly were aligned to the predicted Smp gene sequences from the *S*. *mansoni* genome version 5.2, utilizing BLASTn [17] and applying as threshold an e-value = 10^−5^, coverage ≥ 20% and strand specificity. In the case of the alignment of a contig to multiple Smps, the hit with higher identity was considered as the correct Smp alignment. For each Smp with multiple contigs aligned to it, we considered the contig with the highest coverage of the Smp as the representative of evidence of expression of that Smp.

All Trinity-assembled contigs were aligned to the genome sequence using BLAT [21]. Contigs with genomic coordinates that did not intersect the genomic coordinates of any Smp-predicted gene were analyzed with InterProScan [22] using the Pfam database [23], Batch CD-search tool [24] and Coding Potential Calculator (CPC) [25] to assess the protein-coding potential of these transcripts. Contigs with a Pfam conserved domain were clustered into categories using domain-centric Gene Ontology (dcGO) [26] and the online tool CateGOrizer (http://www.animalgenome.org/tools/catego/) with the GO_slim2 option.

### Gene Family Phylogeny Tree of Lifeguard proteins

Multiple alignments of Lifeguard proteins from *S*. *mansoni*, from five other schistosome species (obtained from ftp://ftp.sanger.ac.uk/pub/pathogens/HGI/) and from various invertebrate and vertebrate species were restricted to the BAX1-I domain and were performed utilizing the ClustalX2 program [27]. Complete sequences for the orthologs with high identity to SmDLFG1 and 2 proteins were obtained from the five other schistosome species by analyzing their preliminary genome sequence with the program Spaln [28]. Except for the latter *Schistosoma* sequences, the accession numbers of the sequences used in the analysis are indicated in Fig G in S1 Text. Phylogenetic analyses were performed using Bayesian inference methodology using MrBayes program v3.2.2 x64 [29]. The analysis was performed using default parameters, except for the use of the command “prset aamodelpr = mixed,” which enables sampling across all fixed amino acid rate matrices (models for amino acid evolution) implemented in the program. Analyses were stopped after 1,000,000 generations, with samplings every 100th generation. Tree information was summarized utilizing “sumt burnin = 2500”, which discards the first 250,000 generations. A measured potential scale reduction factor (PSRF) parameter equal to 1 was obtained using the “sump burnin = 2500” command, indicating a convergence of the analysis. The resulting tree was visualized using the TreeView program [30].

### Differential gene expression among eggs, females and males

Transcript abundance in eggs, females and males was quantified using the Trinity assembly output and the reads from each form as input for the Sailfish tool [31]. The number of reads was normalized using the upper quartile, correcting for the different sequencing depths of the libraries. Significant differential expression between two conditions (egg versus female; egg versus male; female versus male) was computed using the NOISeq program [32] with the NOISeq-sim option and the following parameters: *nss* = 5, to simulate five technical replicates, each comprising 20% of the reads in the dataset (*pnr* = 0.2), allowing a small variability (*v* = 0.02). To identify contigs with significant differential expression, a probability P ≥ 95% was used as the cutoff. Next, with the list of contigs representative of each Smp, we searched for the most highly expressed genes in the egg, the female or the male. For this purpose, we identified genes that simultaneously had a significantly higher expression in eggs in the NOISeq comparison with both males and females, and we repeated the procedure, identifying the genes that simultaneously had a significantly higher expression in females than in both eggs and males, as well as the genes that simultaneously had a significantly higher expression in males than in both eggs and females; these genes were flagged on the full list of significantly differentially expressed genes as Egg_High, Female_High or Male_High, respectively.

The most highly expressed genes in eggs, females or males were categorized using Gene Ontology (GO) terms and the Ontologizer tool [33], with all genes detected in the transcriptome as background. GO terms for *S*. *mansoni* genes were obtained from the Metazoa Mart database (http://metazoa.ensembl.org/biomart/martview/), and *p-values* were calculated by the parent-child union method.

### Refinement of gene models

To refine the *S*. *mansoni* predicted gene model structures, we mapped our Trinity-assembled contigs to the genome using BLAT [21]. We then used Bedtools utilities [34] to cross-reference the mapped coordinates of our RNA-Seq transcripts with the coordinates of the coding sequences of the Smp predicted genes [4] and flagged the Smp predicted genes that were extended at the 3′-end and/or the 5′-end. Moreover, we flagged genes in the vicinity of one another that were merged by our RNA-Seq data. Next, we compared our list of extended genes obtained above with the 5′- and 3′-UTR annotations available for 2,160 Smp genes [4] (excluding the UTR annotations of another 617 Smp genes for which the UTR coordinates are inconsistent with the coordinates of the coding sequence), and we flagged the Smps for which our assembled contigs predicted a different or a longer UTR.

To assess further evidence for the transcription start site (TSS) of *S*. *mansoni* genes, we downloaded the Chromatin Immunoprecipitation Sequencing (ChIP-Seq) dataset of adult worm Histone H3K4me3 (SRR1107840) [9], and we used the HOMER pipeline [35] to map the ChIP-Seq sequences to the genome and to find the genomic coordinates of H3K4me3 enriched peaks. HOMER found enriched peaks by calculating the density of the reads at the peaks that should be at least 4-fold higher than the peaks in the surrounding 10 kb region [35]. We then used Bedtools with a window of ± 500 bp to search for an H3K4me3 peak around the Smp gene 5′-end or around the RNA-Seq transcript 5′-end. We flagged the RNA-Seq transcripts that extended the 5′-end of Smp genes and had an H3K4me3 peak around this new 5′-end. Using the Bedtools closest function, we compared all expressed Smp genes of males and females having the H3K4me3 mark near (within +/- 500 bp of) the 5´-end against the dataset of expressed Smp genes without the histone mark to evaluate the prediction of gene TSS.

### *S*. *mansoni* genome browser

Our gene models (Trinity-assembled contigs) and all the histone mark data [9] mapped to the genome are accessible through a local installation of the UCSC Genome Browser in a Box (GBiB) [36] at http://schistosoma.usp.br/.

### Validation of differentially expressed genes by real-time RT-PCR (qPCR)

Total RNA was extracted from eggs using Trizol reagent (Invitrogen), according to the manufacturer's instructions, and treated with DNAse I using the RNeasy Micro Kit (QIAGEN, Germantown, MD, USA). RNA from adult female and male worms was extracted and treated with DNase I using the RNeasy Mini Kit (QIAGEN). Three biological replicates were assessed for each life cycle stage and form. The Reverse Transcriptase (RT) reaction was performed with 1.0 μg of each total RNA sample using the SuperScript III First-Strand Synthesis SuperMix (Invitrogen). Each real-time qPCR was run in three technical replicates with Sybr Green PCR Master Mix (Applied Biosystems, Thermo Scientific, USA), 160 nmol of each primer (Forward and Reverse) and cDNA from the reverse transcription, using the 7500 Real-Time PCR System (Applied Biosystems) with the default cycling parameters. Specific pairs of primers (S1 Table) for selected genes were designed using the Primer 3 tool (http://biotools.umassmed.edu/bioapps/primer3_www.cgi) with default parameters, anchoring each primer of a pair on a different exon. The housekeeping gene PAI1 (Smp_009310) was chosen from nine genes that showed no differential expression in the RNA-Seq data, and the real-time qPCR data for the nine genes is shown in Fig B in S1 Text. Data were analyzed using the RefFinder tool [37] to determine a geometric mean of ranking values for each gene among the three stages and to choose the most stable gene for qPCR normalization (lowest ranking gene). Real-time data were normalized in relation to the level of expression of the PAI1 gene, and *p-values* were determined by one-way analysis of variance (ANOVA) and Tukey post-hoc tests. The statistical significance of the correlation between the RNA-Seq and qPCR data was calculated using the Spearman test.

### Ethical statement

Infected hamsters were maintained at the Instituto Adolfo Lutz, and the Comissão de Ética no Uso de Animais do Instituto Adolfo Lutz (CEUA-IAL) reviewed and approved the animal care and use protocol, license number 07/2013. The experimental procedures were conducted according to the Brazilian national ethical guidelines for animal husbandry (Lei 11794/2008).

## Results

### Transcriptome assembly from egg, female and male RNA-Seq data

We performed separate sequencing of dscDNA strand-oriented libraries generated using RNA transcripts of seven-week-old *S*. *mansoni* adult male or female worms recovered from hamster portal vein perfusions and of *S*. *mansoni* eggs recovered from hamster livers. A total of ~2.6 million RNA-Seq reads was obtained, and reads matching transposon, mitochondrial and ribosomal genes were filtered out, resulting in ~1.5 million high-quality, strand-oriented, long reads, with an average length of 278 nt (ranging from 40 to 1,026 nt) (Table 1). Each of the three parasite forms was sampled (250 to 730 thousand reads each, Table 1); however, the egg sequencing depth was hampered because the RNA yield, purity and stability were lower than those of the adult worms.

**Table 1:** Summary of the strand-oriented RNA-Seq data for *S*. *mansoni* eggs and female and male adult worms.

Sequencing results			
Total sequenced reads		2,568,188	
Total filtered reads used for the transcriptome assembly		1,495,134	
	Egg reads		252,383
	Female reads		733,589
	Male reads		509,162
Average read length (min-max length)		278 nt (40–1,026 nt)	

Using the Trinity *de novo* assembler [20] without a reference genome, we obtained 23,967 contigs representing the *S*. *mansoni* transcriptome from eggs and adult worms, with an average contig length of 669 nt (ranging from 201 to 6,508 nt) (Table 2). Of this total, 3,799 contigs represented different isoforms (Table 2) assembled by Trinity that belonged to 1,676 putative alternatively spliced transcript fragments, i.e., an average of 2.3 isoforms per contig. The remaining 20,168 contigs corresponded to unique transcript fragments, with no evidence of alternative splicing (Table 2).

**Table 2:** Summary of the strand-oriented RNA-Seq *de novo* assembly for *S*. *mansoni* eggs and female and male adult worms.

*De novo* assembly results			
Total number of Trinity contigs		23,967	
	Contigs from genes with no alternative splicing		20,168
	Contigs of putative alternatively spliced isoforms		3,799
# of contigs matching known *S*. *mansoni* Smp genes		13,268 (55% of total)	
# of contigs mapping to the genome outside of Smp genes		10,472 (44% of total)	
# of contigs not mapping to the genome		227 (1% of total)	
Average contig length (min-max length)		669 nt (201–6,508 nt)	

We then mapped the contigs to the genome to cross-reference the genomic coordinates with the coordinates of predicted Smp genes [4] and found that 13,268 contigs (55% of total) matched 6,760 known predicted Smp genes (Table 2), an average of two contigs per targeted Smp gene. Notably, a large number of contigs (10,472, 44% of total) had no overlap with any predicted Smp gene (Table 2), and these contigs were found to map to intergenic regions of the genome, to intronic gene regions or to the antisense strands of Smp genes. Less than 1% of contigs (227 contigs) did not map to the genome (Table 2). Some of these contigs might belong to genome sections that have not been sequenced yet.

We subsequently cross-referenced the genomic coordinates of known *S*. *mansoni* retrotransposons [38] with the coordinates of the contigs that mapped outside of Smp genes (intergenic and intronic antisense regions), searching for the intersection of coordinates between them. We found that despite having filtered out individual transposon reads from the input file, the assembly still contained a few contigs (98 out of the 10,472, i.e., 0.9%) that overlapped with retrotransposon regions.

### Differential gene expression in *S*. *mansoni* eggs, females and males

We detected evidence of expression for 6,760 predicted Smp genes (Table 3) by cross-referencing the Smp gene coordinates with the coordinates of contigs that matched those Smps (see S2 Table for the list of genes and their expression levels). Of this total, 4,610 genes were expressed in the egg stage, 6,288 were expressed in females and 4,947 genes were expressed in males (Fig C in S1 Text). Interestingly, 3,443 genes were expressed in all three parasite forms (Fig C in S1 Text).

**Table 3:** Gene expression in *S*. *mansoni* eggs and female and male adult worms, as detected by RNA-Seq.

Expression data summary			
Total *S*. *mansoni* predicted Smp genes expressed		6,760	
	Genes expressed in eggs		4,610
	Genes most highly expressed in eggs relative to males and females		615
	Genes expressed in females		6,288
	Genes most highly expressed in females relative to eggs and males		672
	Genes expressed in males		4,947
	Genes most highly expressed in males relative to eggs and females		510
Putative novel *S*. *mansoni* genes (encoding conserved domains)		232	
	Putative novel genes with coincident H3K4me3 mark		79
Novel transcripts not encoding conserved domains		10,444	
	With protein-coding potential (without identifiable domains)		703
	Putative long non-coding RNAs (lncRNAs)		9,741

**Fig 1:**
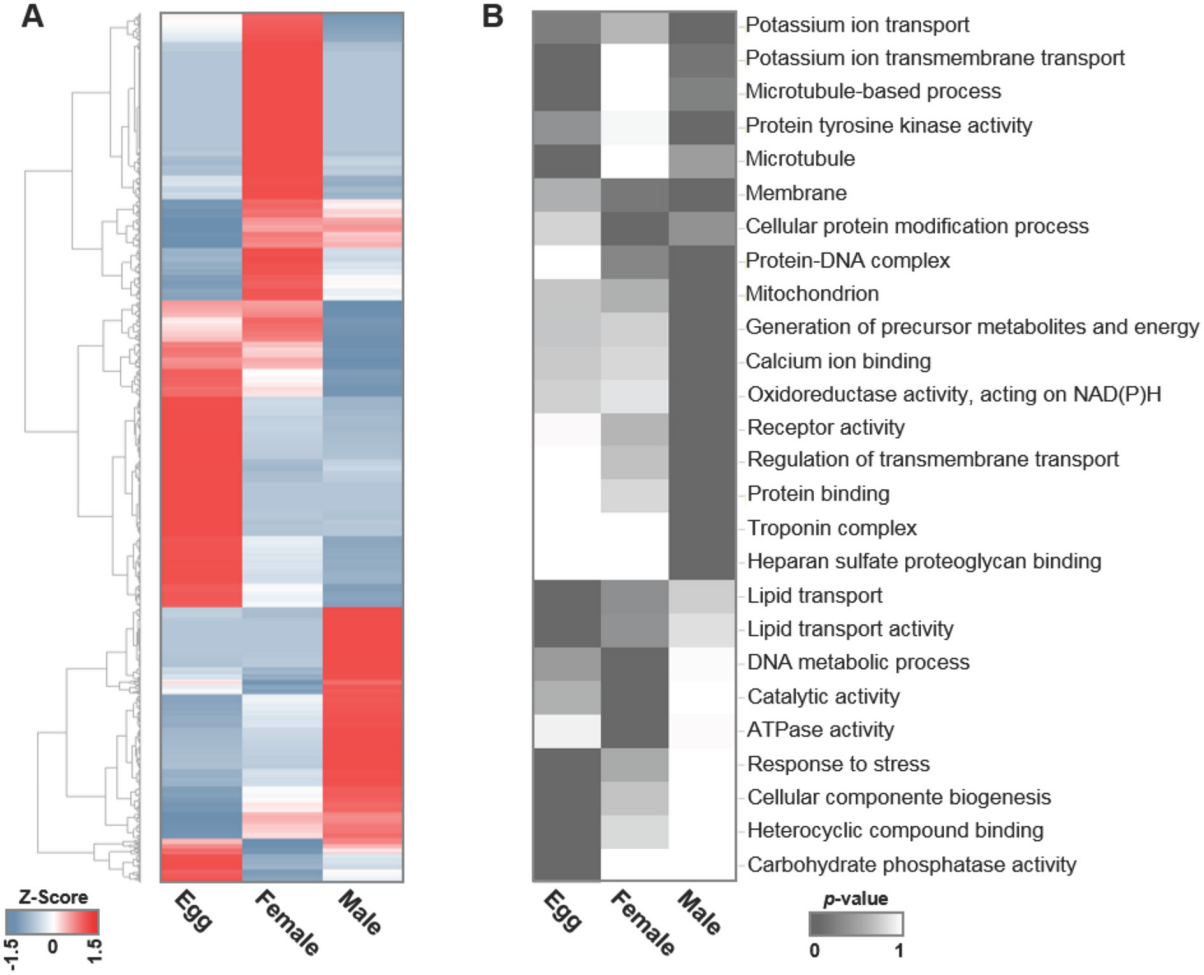
Differential transcriptome profile of *S*. *mansoni* eggs, females and males. (A) Gene expression profile of 4,364 significantly (P ≥ 95%) differentially expressed genes in eggs, females and males. The expression level for each gene (each horizontal line) is normalized among the three parasite forms by the Z-score method; high gene expression is shown in red, and low gene expression is shown in blue. (B) Enriched GO categories of genes most highly expressed in *S*. *mansoni* eggs, females or males. The parent-child union approach was applied, and a *p-value* ≤ 0.01 was considered significant (dark gray). Decreasing shades of gray indicate GO categories that are present but not significantly enriched (*p-value* > 0.01), and white indicates the absence of that enriched GO category in that parasite form.

The quantitative expression level of Smp genes for each parasite form (egg, female or male) was assessed by counting the number of reads from the respective library that matched to each contig, using normalized expression data to correct for the differences in sequencing depth for each dataset. Subsequently, pairwise comparisons between the stages identified the contigs with significant (P ≥ 95%) differential expression among the three conditions; a total of 4,364 contigs were detected as significantly differentially expressed in one stage compared with at least one other stage, and these differentially expressed genes are shown in Fig 1A (see S2 Table for the list of genes and their corresponding differential expression significance).

The number of Smp genes that were most highly expressed in one given form compared with both the other two forms was identified (Table 3), and these genes were flagged as Male_High, Female_High or Egg_High in S2 Table. By this approach, we detected 510 genes most highly expressed in males, 672 genes most highly expressed in females, and 615 genes most highly expressed in eggs (S2 Table). This set of genes corresponds to a representative differential gene expression profile for each parasite form.

Interestingly, we found five micro-exon genes (MEGs) most highly expressed in males, namely two MEG-4 genes (Smp_085840 and Smp_163630), MEG-8 (Smp_171190), MEG-11 (Smp_176020) and MEG-14 (Smp_124000) (S2 Table). In addition, MEG-1 (Smp_122630) was most highly expressed in females, while MEG-5 (Smp_152580) was detected as highly expressed both in females and males. Three MEGs were most highly expressed in eggs, namely two MEG-2 genes (Smp_159810 and Smp_180310) and MEG-3 (Smp_138080).

To identify significantly enriched gene categories among the genes most highly expressed in eggs, females or males, we performed GO analyses. Significantly enriched GO categories (*p-value ≤* 0.01) identified gene groups related to a number of different parasite development and maintenance biological processes (Fig 1B and S3 Table). Some categories were present in more than one parasite form but with a significant enrichment *p-value* in only one. Among the Molecular Function and Biological Process ontologies, the three most significantly enriched GO categories in eggs were carbohydrate phosphatase activity (GO:0019203), lipid transport (GO:0006869) and response to stress (GO:0006950). In females, the enriched categories were cellular protein modification process (GO:0006464), DNA metabolic process (GO:0006259) and catalytic activity (GO:0003824). In males, the three most significantly enriched GO categories were calcium ion binding (GO:0005509), potassium ion transport (GO:0006813) and protein tyrosine kinase activity (GO:0004713).

Taken together, these results point, for the first time, to relevant biological processes enriched in the *S*. *mansoni* egg stage compared with adult worms. In addition, these results indicate a set of genes involved in biological processes enriched in either *S*. *mansoni* male or female worms.

### Validation by real-time RT-qPCR of selected genes most highly enriched in one life cycle form as detected with RNA-Seq data

**Fig 2:**
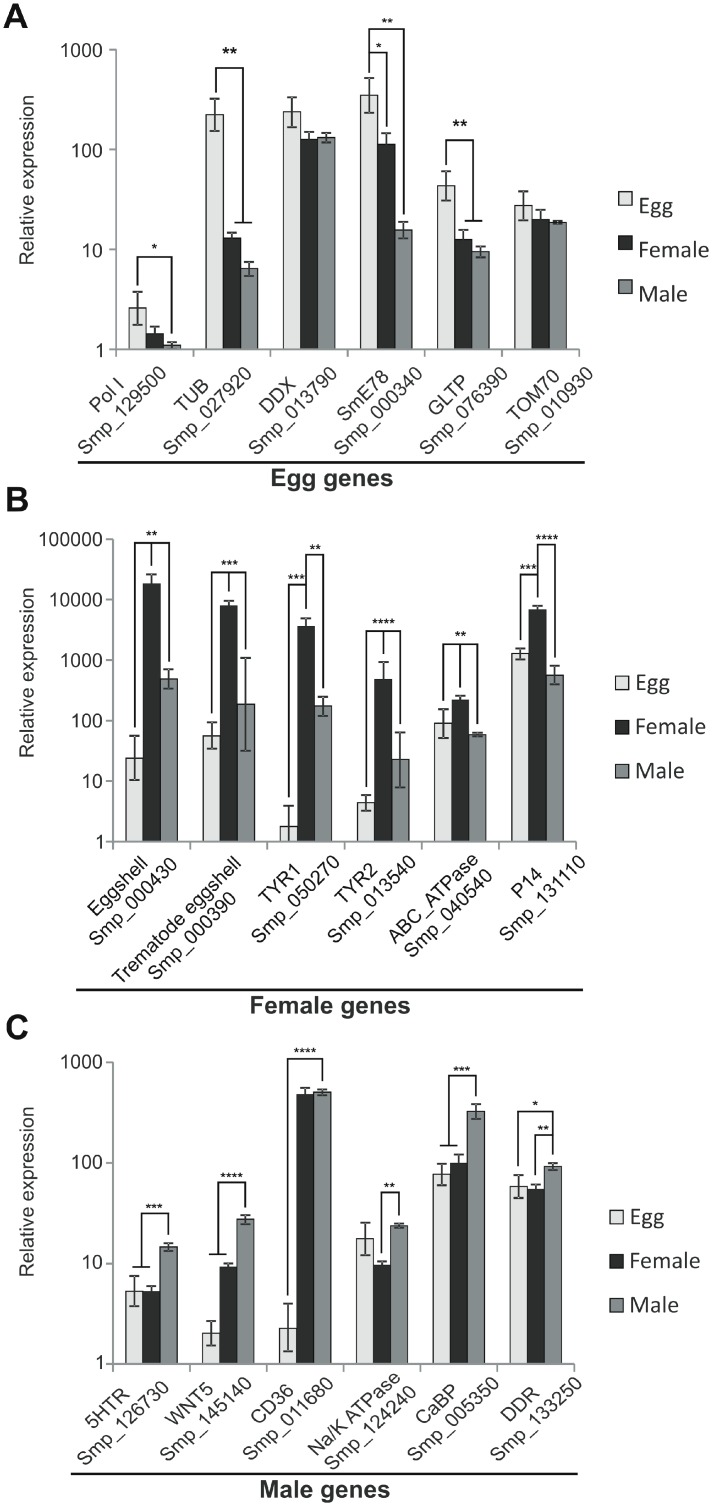
Validation by RT-qPCR of the expression levels of genes detected by RNA-Seq as differentially expressed among the three *S*. *mansoni* forms. Six genes, which were identified as differentially expressed by RNA-Seq, were selected for validation with RT-qPCR for each parasite form, namely (A) egg, (B) female, and (C) male. The lowest biological replicate value of the least expressed gene (Pol I in egg, panel A; Tyr1 in female, panel B; CD36 in male, panel C) was chosen as reference and arbitrarily set to 1, and the expression values of the other biological replicates of the reference gene, and of the other genes, are represented as the relative expression on a log10 scale (y axis). Bars represent standard deviation of the mean for the three biological replicates. The PAI1 gene (Smp_009310) was used for internal normalization among the three parasite stages. The ANOVA Tukey test was used to calculate the statistical significance of the expression differences among the three parasite forms (*p-value ≤ 0.05; **p-value ≤ 0.01; ***p-value ≤0.001; ****p-value ≤0.0001).

In the literature, there is an absence of qPCR data comparing egg, female and male gene expression; similarly, a control housekeeping gene for *S*. *mansoni* has been previously evaluated in the literature only for mixed sex adult worms [7,39]. Additionally, the α-tubulin gene has been used in many studies as a housekeeping gene for normalization among all life cycle stages; however, in our RNA-Seq and qPCR data, α-tubulin is highly differentially expressed in eggs, with approximately 25 times higher expression in this stage when compared with its expression in male and female adults, as detected by qPCR (Fig 2A). In this context, we chose the Plasminogen Activator Inhibitor PAI1 gene (Smp_009310.1) as a housekeeping gene from 9 possible candidates identified by searching the set of genes expressed by all three parasite stages in the RNA-Seq data for the genes that were not differentially expressed in the three RNA-Seq pairwise comparisons of this study. We confirmed PAI1 by qPCR as an adequate housekeeping gene for use in the normalizations by performing qPCR for all 9 candidate normalizer genes and analyzing the data with RefFinder [37], as shown in Fig B in S1 Text.

Using PAI1 as a housekeeping gene, we selected and tested by qPCR six differentially expressed genes for each parasite form to provide for an independent validation of the RNA-Seq data with different biological samples. These genes were selected for their association with important biological functions in each of the parasite forms, as noted later in the Discussion.

We selected glycolipid transfer protein (GLTP), tubulin, translocase of outer membrane 70 (TOM70), RNA polymerase I (PolI), DEAD box RNA helicase (DDX) and nuclear receptor SmE78 as genes highly expressed in eggs. All genes displayed higher expression in eggs when assayed by qPCR, but only four genes were statistically significant when compared with their expression in males and females; in one additional case, only the difference between eggs and males was statistically significant (Fig 2A). The egg RNA-Seq data for the selected genes correlated well with the qPCR data, with a Spearman’s correlation of 0.77 and *p-value* < 0.001.

We selected the tyrosinases Tyr1 and Tyr2, p14, eggshell protein, trematode eggshell protein and ATP-binding cassette transporter as genes highly expressed in females, based on the RNA-Seq data. The qPCR confirmed the higher expression in females compared with that in eggs and males for all six selected genes (Fig 2B), with a Spearman’s correlation between RNA-Seq and qPCR of 0.80 and *p-value* < 0.0001.

We selected Na/K ATPase, calcium binding protein (CaBP), Discoidin domain receptor (DDR), serotonin receptor (5HTR), Wnt5 and scavenger receptor CD36 as genes highly expressed in males, based on the RNA-Seq data. All genes displayed higher expression in males, but this difference was statistically significant when compared with the expression in eggs and females in only four of the genes. In the two remaining cases, the difference was statistically significant only when compared with either females or eggs (Fig 2C). The Spearman’s correlation between the qPCR and RNA-Seq data for males was 0.50, and *p-value* = 0.034.

### Transcripts refining Smp gene structures

RNA-Seq contigs were used to improve the Smp gene model predictions from the 5.2 version of the genome [4], which includes a total of 10,852 Smp gene models. RNA-Seq Trinity-assembled contigs that mapped to the genome with genomic coordinates intersecting the coordinates of gene model exons of any predicted Smp transcribed in the same strand were considered as evidence of an mRNA transcribed from that gene. Using this approach, it was possible to document the extension of Smp genes, and these extensions could occur either at the 5′- or the 3′-end of genes. Our transcriptome data extend the 5′-ends of 3,337 genes and the 3′-ends of 2,417 genes, of which 747 were extended at both ends (S4 Table). It is evident that the majority of genes were extended at the 5′-end, and this bias resulted from using RNA-Seq libraries that predominantly covered the 5′ region of RNAs, as documented by mapping the individual reads along the *S*. *mansoni* complement of genes (Fig D in S1 Text).

Protasio and collaborators [4] had previously improved the original Smp predicted gene models [3] by updating 731 predicted genes that were merged or split. Our transcriptome data showed that despite this gene annotation improvement, there are still an additional 589 gene models that should be altered by merging each predicted sequence with the sequence of the neighboring predicted gene (S5 Table); in each case, we found a single transcript contig that overlaps both neighboring gene predictions. A typical case involves Smp_101670 and Smp_124310, which map to adjacent regions in the genome (Chr_1:5,968,156–6,004,959) and are transcribed in the same strand; with our transcriptome data (contig c7177_g7_i1), it was possible to determine that the two predicted genes are actually part of the same transcript (S5 Table). These new gene models contribute to an improved annotation of the *S*. *mansoni* gene complement.

### Transcription Start Sites (TSSs) of genes corrected by novel RNA-Seq contig evidence

In eukaryotes, it is known that the histone H3 lysine 4 trimethylation (H3K4me3) mark is present in the promoter region of expressed genes, in the vicinity of their Transcription Start Sites (TSSs), and in the 5′-UTR region of genes [40]. Roquis and collaborators performed H3K4me3 chromatin immunoprecipitation followed by sequencing (ChIP-Seq) of mixed-sex adult worms [9], and we used this dataset to assess the presence of enriched peaks of the H3K4me3 mark that could indicate the TSS regions in the *S*. *mansoni* genome. The H3K4me3 peaks were found near (within +/- 500 bp of) 3,084 Smp genes, showing that these gene models have their predicted 5′-end close to the promoter region (S4 Table). Among these genes, 2,454 genes have a contig from our dataset confirming the 5′-end of the Smp gene. With the extension of the 5′-end of another 3,337 Smp genes (that were re-structured using our RNA-Seq data), the H3K4me3 TSS histone mark was identified for a further 673 re-structured Smp genes (S4 Table).

Using all expressed genes found in our RNA-Seq data, we searched for the closest H3K4me3 histone mark around the predicted Smp annotation TSS regions or the extended Smp TSS regions. Unsurprisingly, for those expressed genes for which we found an H3K4me3 histone mark near the TSS (+/- 500 bp from the TSS), we detected a peak centralized at the midpoint of the TSS, but also a tail extending downstream from the peak (Fig E in S1 Text, blue line). For those expressed genes without the histone mark at the gene TSS (no H3K4me3 within +/- 500 bp from the TSS), the closest H3K4me3 was found approximately 1–2 kbp upstream of the TSS or 1 kbp downstream of the TSS (Fig E in S1 Text, red line). These distance distribution patterns are different from the distribution pattern expected by chance, obtained in a random-position-generated TSS set (Fig E in S1 Text, gray line).

**Fig 3:**
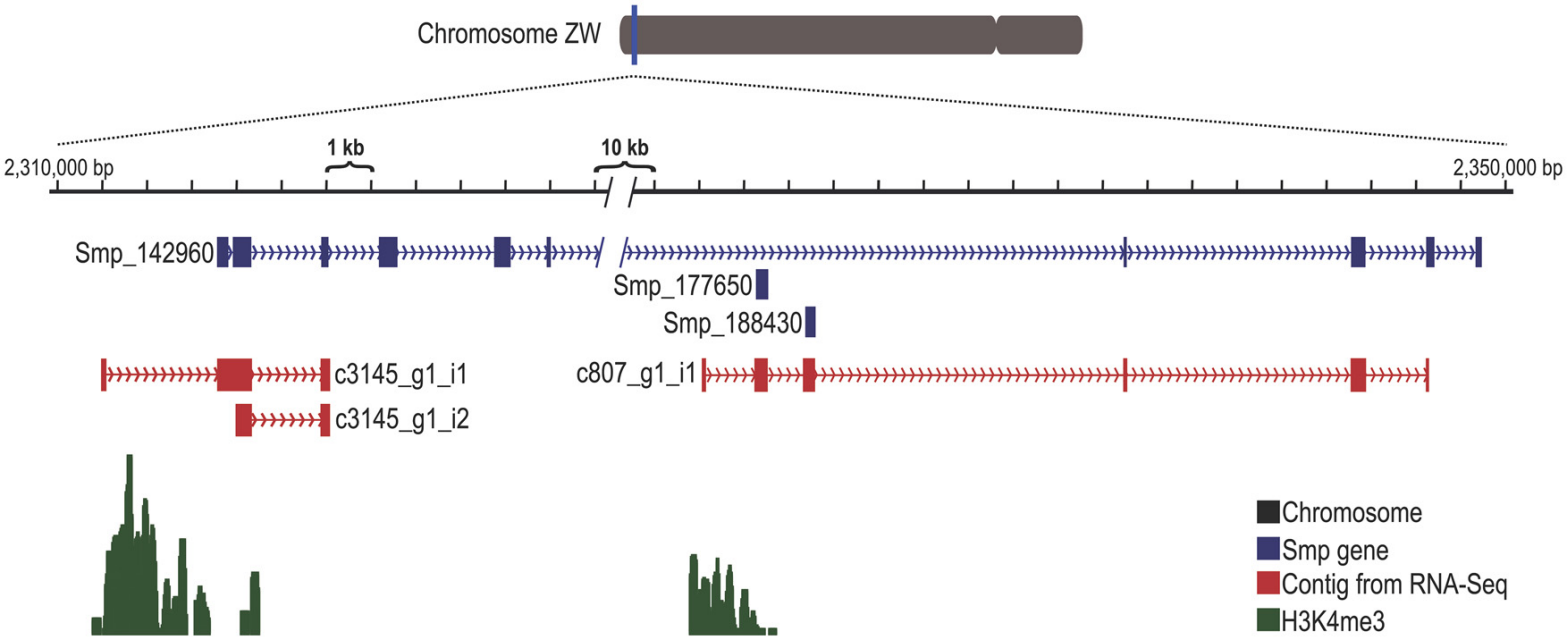
Contigs from the RNA-Seq data improve the Smp gene predictions, including the rearrangement of gene structure and the addition of new 5′-ends. The figure shows a Chromosome ZW 40-kb locus (Chr_ZW:2,310,000–2,350,000) harboring Smp_142960, Smp_177650 and Smp_188430 (blue). Contigs (red) from our RNA-Seq assembly align to Smp genes, showing their new architecture. The new contig c807_g1_i1 (red boxes on the right-hand side) merges Smp_177650 and Smp_188430 with the final exons from Smp_142960 and has a new 5′-end exon that overlaps the histone H3K4me3 peak (green). The new contig c3145_g1_i1 (red boxes on the left-hand side) matches the first two exons of Smp_142960 and adds a new 5′-end exon that corrects the TSS of the gene and overlaps the histone H3K4me3 peak (green). This locus can be seen at http://schistosoma.usp.br/ by entering the chromosome coordinates, the Smp number or the contig number in the “*enter position or search terms*” field at the top of the browser.

To exemplify the scenario of improved Smp gene models, we selected a specific *S*. *mansoni* locus (Chr_ZW: 2,310,000–2,350,000), which hosts the Smp_142960 gene (Fig 3, blue boxes and arrowheads) and two additional short mono-exonic genes, namely Smp_177650 and Smp_188430 (Fig 3, short blue boxes within intron 6 of Smp_142960). Our RNA-Seq assembly generated three contigs that map to the locus with a completely different architecture (Fig 3, red boxes and arrowheads); all three contigs new 5’-ends coincided with H3K4me3 histone marks (Fig 3, green peaks), confirming that they represent new genes with novel TSSs. The new gene on the upstream side of the locus (contig c3145_g1_i1) encodes an isoprenoid biosynthesis enzyme, Class 1 domain (cl00210) (e-value = 1.92∙10^−6^), and is not part of the glutathione synthase gene originally annotated as Smp_142960. In fact, the full domain of glutathione synthase is encoded by the new gene (contig c807_g1_i1) on the downstream side of the locus. It should be noted that the 3′-end of both these new genes was not covered by our RNA-Seq data, which is consistent with the fact that our new RNA-Seq method covered predominantly the 5′-ends of genes (Fig D in S1 Text).

This analysis cross-references ChIP-Seq information on TSS histone marks with RNA-Seq contig data and looks for evidence of an extended Smp gene prediction, where the 5′-end of the gene overlaps the TSS histone mark, and is shown here for the first time for *S*. *mansoni*, adding important information regarding the parasite’s gene regulatory regions. Using these data, it was possible to observe that the histone H3K4me3 deposition in *S*. *mansoni* frequently overlaps the first exon of the gene and extends into the first intron (Fig E in S1 Text).

### Novel *S*. *mansoni* transcripts—new protein coding genes

The large number of contigs (10,472 contigs) with no match to Smp genes raised the hypothesis of potential new genes not yet described in *S*. *mansoni*, and transcripts with open reading frames (ORFs) longer than 150 nt were considered as candidates for further analyses. Subsequently, using the Conserved Domains Database (CDD), we investigated the similarity of the proteins encoded by these ORFs with protein-conserved domains present in proteins from other species, which identified 232 contigs encoding protein-conserved domains, thus indicating potential new genes (S6 Table), among which 159 contain full-length ORFs. These contigs were deposited at NCBI TSA under accession number GDQY01000000. For the entire set of potentially new protein-coding genes, we assessed the presence of an H3K4me3 TSS histone mark close to the 5′-end of the contigs and found 79 with evidence of this mark in the gene promoter region (S6 Table).

In this set of potential new *S*. *mansoni* genes, we searched for contigs encoding protein-conserved domains not yet reported among the Smp genes. Closer inspection revealed contigs encoding significantly conserved protein domains (score < 1 x 10^−5^, covering approximately 70 to 100% of the domain), none of which were present in any Smp gene (S6 Table).

**Fig 4:**
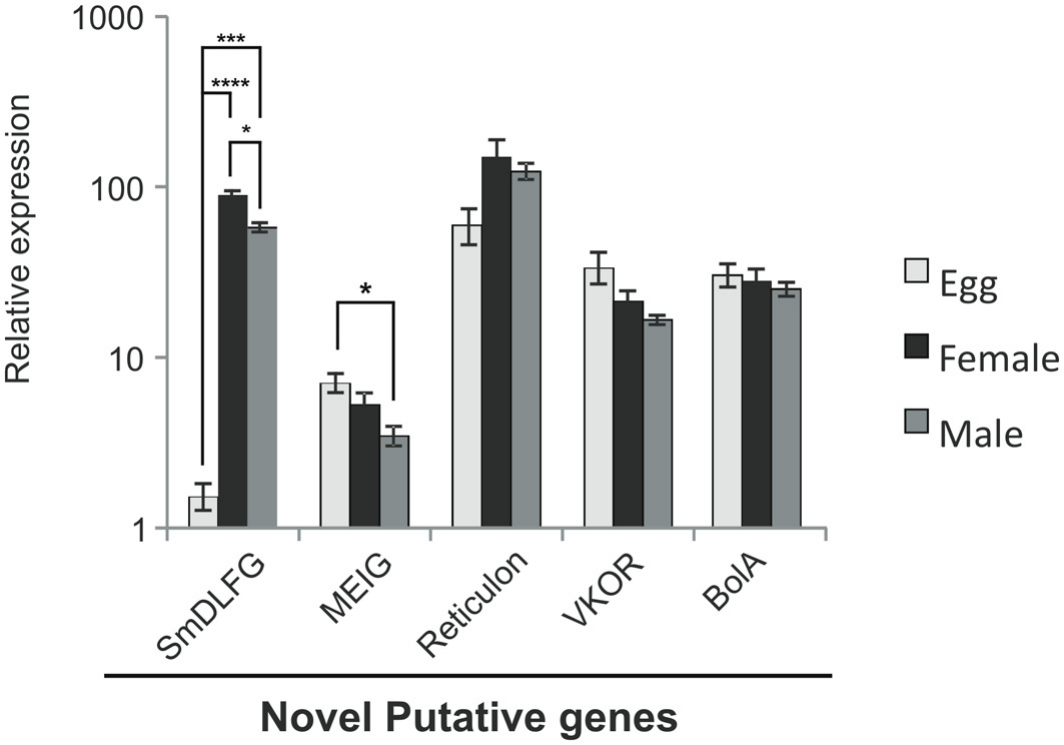
Relative expression obtained by qPCR of new putative genes identified in the RNA-Seq data of *S*. *mansoni* eggs, females and males. The expression level of one of the three biological replicates (with lowest expression level) of the SmDLFG gene in the egg stage was chosen as reference and arbitrarily set to 1, and the values for the other two replicates of the reference gene, and for the other genes and stages, are represented as fold changes relative to this value on a log10 scale (y axis). Bars represent the standard deviation of the mean for the three biological replicates. The gene PAI1 (Smp_009310) was used for internal normalization among the three parasite forms. The ANOVA Tukey test was used to calculate the statistical significance of the differences in expression among parasite forms (*p-value ≤ 0.05; ***p-value ≤ 0.001; ****p-value ≤ 0.0001).

Specifically, our RNA-Seq data identified an interesting new putative gene with a meiosis expressed gene (MEIG) protein-conserved domain, which was not predicted among Smp genes. This gene plays an essential role in the regulation of spermiogenesis in mammals [41] and was also detected in ovaries of mouse embryos when the oocytes reached the prophase I meiotic stage [42]. The SmMEIG gene encodes a protein with 85 amino acids and is orthologous to the *S*. *japonicum* MEIG gene (accession number CAX73271.1, identity = 91% and e-value = 6∙10^−50^, covering 100% of the target gene), as determined using BLASTx with the nr NCBI protein database. This putative gene was only detected in male RNA-Seq data, but the qPCR results (Fig 4) showed that this new gene is expressed in all three parasite stages; the expression in eggs is two-fold higher than that in males. This gene could play an important role in schistosome female oocyte and male sperm production, and the higher expression in eggs suggests a function not yet characterized in this parasite stage.

Another new putative gene encodes the enzyme VKOR, which is responsible for the recycling of vitamin K cofactor in eukaryotes, reducing vitamin K that is oxidized upon the carboxylation of glutamic acid residues of proteins in apoptosis, signal transduction and growth control pathways [43]. This new putative gene is expressed in eggs, females and males, as detected by RNA-Seq data and qPCR (Fig 4), showing that the parasite might use this cofactor in the metabolic pathways in all three parasite forms. This putative VKOR gene is found in many mammal species, and using the BLASTx tool, we found that the *S*. *mansoni* VKOR gene is orthologous to the *H*. *sapiens* gene with an identity of 30% and e-value of 10^−12^, covering 41% of the target gene.

We selected other new putative genes detected in *S*. *mansoni* by RNA-Seq for testing with qPCR, such as Reticulon and BolA. These new putative genes were detected in all three parasite forms (Fig 4), confirming their expression. Reticulon proteins in eukaryotes are localized to the endoplasmic reticulum, and there is evidence that they influence endoplasmic reticulum-Golgi trafficking, vesicle formation and membrane morphogenesis [44]. The BolA gene is widely conserved from prokaryotes to eukaryotes and seems to be involved in cell proliferation or cell-cycle regulation [45]. We confirmed the presence of these putative new genes in the three parasite forms.

We also detected a new *S*. *mansoni* gene from the transmembrane BAX inhibitor motif (TMBIM) family containing the Bax Inhibitor 1 domain (BI-1) [46]. This new protein-coding gene (contig c17331_g1_i1) encodes a 259-amino-acid-long protein that shares similarity with the Lifeguard members of this family, with an expected value of 0.003 (27% identity and 43% similarity) for the alignment with a *Drosophila wilistoni* protein containing a BAX1_i domain. Several other hits with similar proteins containing this domain are obtained, confirming the consistency of this result. Indeed, we constructed a PSSM matrix with the new protein utilizing PSI-blast and considering the proteins aligned with e-value cutoff of 0.01; after a single round of iteration using this PSSM matrix, at least one hundred different Lifeguard proteins were aligned with e-values lower than 10^−30^. This result indicates that although this new protein displays a relatively divergent sequence from known Lifeguard proteins, it retains residues that are conserved among members of this family. Moreover, a comparison between the transmembrane helix profile of this protein and of a known Lifeguard protein revealed a very high similarity, providing further evidence of homology between the sequences (Fig F in S1 Text). Therefore, we named the new gene SmDLFG1 (*Schistosoma mansoni* Divergent Lifeguard 1).

The RNA-Seq contig c17331_g1_i1 encoding the SmDLFG1 gene does map to two different and adjacent loci on Chromosome 1, in a region where no gene had been predicted. The first higher-score match (BLAT score = 888) is at the Chr_1:623,131–636,930 locus. The second match has some mismatches at the 5′-end of the contig and a lower matching score (BLAT score = 790). Using the genome sequence at the locus of the SmDLFG1 second match (Chr_1:606,203–612,641) and RNA-Seq data from NCBI SRA, it was possible to detect reads with a 100% match, which confirmed the expression of an isoform of SmDLFG1 that we named SmDLFG2, mapping to Chr_1:605,936–612,714.

The five Smp paralog genes from this protein family with complete BAX1_ domain (Smp_044000 or G4VD38, Smp_181470 or G4VEN1, Smp_150500 or G4VGF8, Smp_026160.1 or G4VKQ6, Smp_210790 or G4V7Q6) and the newly identified SmDLFG1 and SmDLFG2 protein-coding genes were used in a phylogenetic analysis that included representative proteins of this family from five other schistosome species as well as from various invertebrate and vertebrate species (Fig G in S1 Text).

RNA-Seq detected the expression, in the three parasite forms, of all six Smp predicted gene paralogs of the TMBIM family, but apart from the SmDLFGs, only Smp_026160 (annotated as Putative growth hormone inducible transmembrane protein) exhibited a significantly higher expression in eggs compared with males or females. The new SmDLFGs are highly expressed in females compared with males and eggs (Fig 4) (the qPCR primers did not distinguish between the two isoforms).

**Fig 5:**
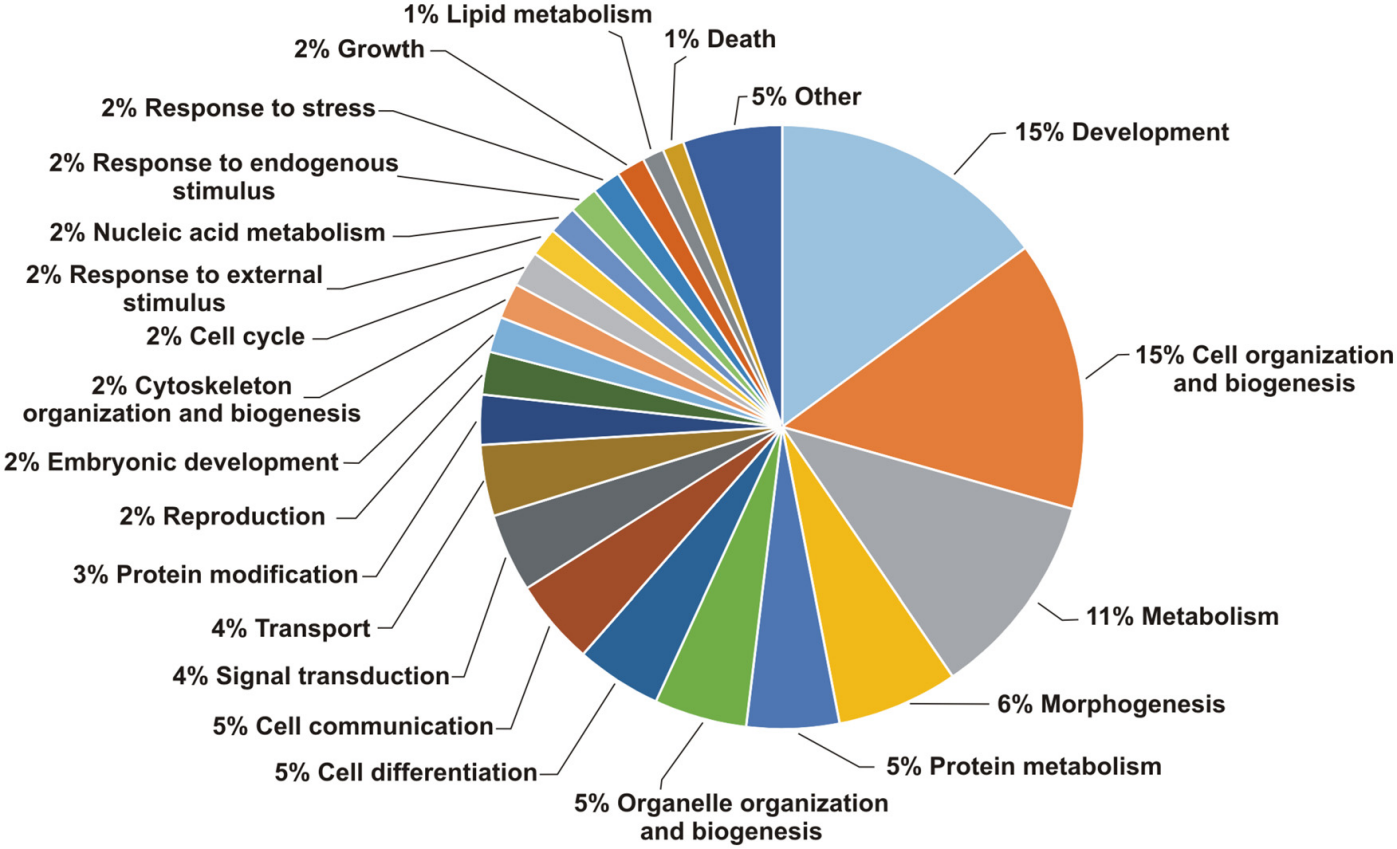
Gene Ontology classification of Pfam conserved domains found in 198 novel protein coding genes. The percentage of Pfam domains in each of the biological process categories of GO categorization was determined. A total of 232 new protein-coding genes presenting 152 different Pfam domains were assigned to 208 biological processes by the dcGO tool, and with the CateGOrizer tool it was possible to categorize GOs by single counting. Categories with less than 1% representation were grouped together.

GO categorization of 198 out of the 232 protein-coding putative new genes, encoding conserved-protein domains, identified GO biological processes related to development, metabolism, cell organization and biogenesis (Fig 5). This dataset of putative new genes encoding conserved-protein domains provides an opportunity of identifying new important genes in metabolic pathways where steps remain missing.

The remaining 10,444 contigs not encoding conserved protein domains were classified according to their protein-coding potential with the CPC tool, which identified only 703 contigs with protein-coding potential (however with no identifiable conserved domain), whereas 9,741 contigs were classified as non-coding and therefore represented putatively expressed long non-coding RNAs (lncRNAs) (S6 Table).

## Discussion

The *S*. *mansoni* genome and transcriptome have been explored for the past decade [47], providing information related to the gene expression profiling and transcription regulation of certain life-cycle stages of the parasite. In this study we have obtained, for the first time, large-scale RNA-Seq separate profiles of *S*. *mansoni* females and males, as well as the egg-derived expression profile. We also used, for the first time, a combination of *de novo* transcriptome assembly with existing genome coordinates of predicted genes, along with newly mapped public ChIP-Seq data, which permitted the identification of novel putative *S*. *mansoni* genes. Because we have, for the first time, generated an individual gene profile for each of these three parasite forms, we explored this additional information by searching the literature for the possible functions of a selected set of genes most highly expressed in each form, as described below.

A set of 6 genes for each of the three parasite forms was selected for validation by RT-qPCR based on the fact that most of these genes were highlighted by the functional analyses mentioned above. Twenty-nine of the thirty-six comparisons (81%) performed confirmed, using RT-qPCR, the significant expression enrichment previously determined by RNA-Seq (Fig 2), a fraction of the confirmation similarly found in the literature [48]. The Spearman correlations between RNA-Seq data and RT-qPCR were in the range 0.50 to 0.80 with *p-values* in the range 0.034 to < 0.0001. We consider this result to be a successful validation, especially if we note that none of the directions of enrichment in the RNA-Seq and RT-qPCR data showed a conflicting opposite result; it is already known that all transcriptome techniques, including microarray, RNA-Seq and qPCR, have inherent pitfalls that affect quantification and cannot be fully controlled [49].

First, we analyzed the differentially expressed genes in the egg stage in light of the known biological characteristics of the eggs. The glycolipid transfer protein (GLTP) (Smp_076390) from the lipid transport GO category is highly expressed in eggs compared with males and females. GLTP is responsible for the intermembrane transfer of lipids linked to sugars, such as glycosphingolipids [50]. As schistosomes are unable to synthesize fatty acids, the uptake of these compounds from the host is essential. We suggest that the enrichment of lipid transport genes in the egg stage might be related to an important uptake of lipids from the host, possibly related to embryo development. In fact, it is known that the schistosome egg uptakes cholesteryl ester from HDL vesicles, mediated by the CD36 receptor, and that this uptake is important for egg maturation [51]. In this context, we speculate that because the CD36 receptor has such an important function in egg development, the enrichment of lipid transport genes among the highly expressed egg genes could be related to the transport function. Thus, GLTP could be an important gene for egg development and maintenance inside the host circulation until the eggs reach the host intestine lumen, where they are released.

Egg formation is dependent on structural components such as microtubules, an enriched GO category. An important gene from the microtubules category is tubulin (Smp_027920), whose expression was 17-fold greater in eggs compared with that in females and 34-fold greater than that in males. Microtubule genes enriched in eggs will integrate the layer between the eggshell and the developing miracidium, known as Reynolds’ layer, comprising microfibrils in a granular matrix [52].

Genes related to stress response were also increased in eggs, and in this group, we highlight the mitochondrial genes from the translocase of outer membrane (TOM) machinery that serve as the main entry gateway of preprotein into the mitochondria [53]. The mRNA expression of TOM70 (Smp_010930) and of HSP70 (Smp_065980) was detected in eggs, and the proteins encoded by these genes are key to the mitochondrial import pathway [54] and contribute to the folding and refolding of proteins after stress denaturation [55].

The mRNA level of Pol I (Smp_129500), which encodes the enzyme responsible for the transcription of ribosomal DNA (rDNA) [56], is significantly higher in eggs than in males, suggesting ribosome production and cellular proliferation inside the egg. Another gene with a high expression level in eggs encodes a protein related to transcription, namely the DEAD box RNA helicase (Smp_013790). DEAD box proteins unwind short duplex RNA and remodel RNA-protein complexes, and they are important players in RNA metabolism from transcription and translation to mRNA decay [57]. The nuclear receptor SmE78 gene (Smp_000340) also showed an increased expression in eggs. Wu et al. had previously shown that nuclear receptors are important transcriptional modulators, and in *S*. *mansoni*, SmE78 may be involved in growth and vitellogenesis [39].

To further validate the gene enrichment analysis of the egg stage, which has the lowest RNA-Seq coverage, we have manually inspected three genes known to be highly expressed in eggs, namely omega-1 [58], IPSE/alpha-1 [59] and kappa-5 [60]. Only the omega-1 and IPSE/alpha-1 genes are annotated as Smp genes, namely Smp_193860 and Smp_112110, respectively. Indeed, they are listed in S2 Table as highly expressed genes in the egg stage with the flag Egg_High, as these two genes were detected in our RNA-Seq data as significantly differentially expressed in eggs compared with males and females.

Interestingly, the omega-1 gene (Smp_193860), which encodes a protein with 127 amino acids, appears in our RNA-Seq data as contig c7716_g1_i1 with an additional five new exons at the 5’-end of the gene, and the new longer ORF encodes an omega-1 protein with 236 amino acids, which is now compatible with its described molecular weight of 31 kDa [58].

The kappa-5 gene with accession AY903301.1 [60] is not yet annotated as an Smp gene; only a paralog, Smp_150240 is annotated in the genome, with an identity of 89% and query coverage of 74% compared to the kappa-5 gene. Because the kappa-5 gene is not present in the Sanger Genome annotation, we did not detect it in the automated analysis, but by a curated manual investigation, using the NCBI accession AY903301.1, it was possible to align the sequence to the genome with genomic coordinates Chr_3:13873256–13879312 minus strand; we found that contig c6481_g1_i1 is the transcript corresponding to the kappa-5 gene. This contig is highly expressed in the egg stage, with normalized egg read counts of 1,909.35 and no reads detected in the adult male and female forms.

Subsequently, we analyzed the genes differentially expressed in females in light of the known biological characteristics of the females. Their transcriptome profile is highly linked with egg production, as the *S*. *mansoni* female produces approximately 350 eggs daily, and proteins that are important to the eggshell structure should be expressed. The hardened and tanned structure of the eggshell is derived from tyrosinase activity, which catalyzes the cross-linking of proteins known as quinone tanning [61]. The mRNA expression levels of Tyrosinase 1 (Smp_050270) and Tyrosinase 2 (Smp_013540) in females are higher than in eggs and males. Interestingly, Tyr1 has a much higher expression level than Tyr2, although the two genes encode highly similar proteins. The higher abundance in females is consistent with the fact that tyrosinase originates from the female vitelline cells inside the vitellaria, acting on eggshell formation.

The most studied *S*. *mansoni* eggshell proteins are p14 and p48. Chen et al. showed that the p14 gene is expressed only in mature female vitelline cells and is undetectable in the RNA obtained from eggs [62]. Our RNA-Seq results show that the p14 (Smp_131110) transcript was detected at low levels in eggs extracted from hamster liver, and the low abundance of this transcript in eggs was confirmed by qPCR. A high expression level of p14 was detected in females compared with males or eggs, as expected. Two other proteins that were not yet shown as part of eggshell synthesis are eggshell protein chorion (Smp_000430) and Trematode eggshell protein (Smp_000390), both with the Trematode eggshell domain (Pfam:08034). We found that the genes encoding these two proteins are highly expressed in females, and compared with p14, the expression of eggshell protein chorion gene is twice as high, pointing to a new protein for possible exploration as a candidate antigen in liver granuloma formation.

Female reproduction requires a high bioenergetic supply for the production of hundreds of eggs per day. Consistently, genes from the ATPase activity category (Fig 1B) are enriched in our analyses of female highly expressed genes, including genes related to the production of ATP through mitochondrial oxidative phosphorylation. Additionally, the ATP-binding cassette transporter gene (Smp_040540) exhibited higher expression in females than in males, but interestingly, the expression level in eggs was almost the same as that in females. In addition to the energy supply for egg production, it was recently proposed that enzymes present in the schistosome tegument could act similarly to human cell-surface ATPases, inhibiting platelet activation and modulating the host coagulation mechanism [63,64].

Finally, we analyzed the differentially expressed genes in adult males in light of the known biological characteristics of the males. Their transcriptome was already individually explored by RNA-Seq [6]; however, the different transcriptome profiles of the two sexes that reflect their distinct biological characteristics were not previously compared. We found that a number of genes most highly expressed in males are involved in the regulation of transmembrane transport (GO:0034762), membrane (GO:0016020) and heparin sulfate proteoglycan binding (GO:0043395), probably belonging to the male tegument. This result is consistent with the fact that the male body is more highly exposed to the host immune cells in the circulation than the female, and consequently, the tegument renewal rate is higher in the male than in the female [65].

Among the most highly expressed genes in males, we found an enrichment of the potassium ion transport category (GO:0006813), and the high expression of the sodium-potassium ATPase gene (Smp_124240) in males compared with in females was confirmed by qPCR. The surface plasma membrane is involved in nutrient uptake, involving several amino acid and sugar transporters, aquaporins, anion selective channels and Na+/K+ and Ca2+ ATPases [66,67].

We detected enriched GO categories among male genes associated with the schistosome muscle layer, such as the troponin complex and the calcium binding protein Sm20 gene (Smp_005350), whose expressions were higher in males than in females, according to both the RNA-Seq data and qPCR. Our RNA-Seq data showed that Smp_005350 is fully transcribed; this gene encodes a 58-kDa calcium binding protein with distinct Ca2+ binding motifs. It should be noted that Mohamed et al. studied a distinct 20.8 kDa antigenic *S*. *mansoni* calcium binding protein encoded by the U91941 cDNA clone and named Sm20.8 [68], subsequently renamed Smp_086530, which is distinct from the Sm20 gene (Smp_005350) detected here as abundant in the male.

Receptor activity and protein tyrosine kinase activity were GO categories enriched in male-expressed genes, such as Discoidin domain receptor DDR (Smp_133250), Serotonin receptor 5HTR (Smp_126730) and Wnt5 (Smp_145140), encoding a signaling molecule. This finding suggests that a characteristic cell signaling process might operate in the male.

The scavenger receptor CD36 antigen transcript (Smp_011680), which is a mediator of cholesteryl ester uptake by adult worms [51], was detected by qPCR in all three forms, with males and females having the same level of CD36 expression, in disagreement with Fitzpatrick et al., who detected the CD36 antigen transcript only in female worms [7]. The divergent result from Fitzpatrick et al. could be explained by the different parasite strain (Puerto Rican strain in the Fitzpatrick et al. study, BH strain in this study).

Micro-exon genes (MEGs) constitute a large family characterized in the parasite genome, each gene with multiple symmetrical exons, arranged in tandem, with lengths that are a multiple of three nucleotides (from 6 to 36 nucleotides for each exon) [3,69]. This arrangement leads to protein variation through alternative splicing [69], and may have an impact on the escape of parasites from host defenses. Differential expression of MEGs between males and females has only been observed with microarrays in a comparison of male esophagus with female gastrodermis [70]. Here we compared eggs, female and male adult worms and identified stage-specific MEGs, most highly expressed in each of the three forms, supporting the idea that MEGs might be specifically modulated in response to defenses of the host. Interestingly, MEG-4 and MEG-14, most highly expressed in males, have been previously described using whole mount *in situ* hybridization to be specifically expressed in the esophagus of adult worms from both sexes [71]. Male and female highly expressed MEG-5 had its protein product previously detected in tegumental preparations [69]. Egg highly expressed MEG-2 and MEG-3 protein products were detected in egg secretions and only the latter was also detected in schistosomula secretions, being produced in the head gland [69]. These observations suggest that either the expression of esophageal MEGs is more robust in males than in females or that in the males this organ contributes with a higher amount of mRNA for the total pool.

We used the transcriptome data as a guide to improve Smp gene predictions, adding UTRs or new coding exons to hundreds of Smp genes. Moreover, we cross-referenced the genomic coordinates of the genes with the genome-wide coordinates that we obtained by genome-mapping the publicly available H3K4me3 adult worms ChIP-Seq dataset [9], and we confirmed the recently described presence in adult schistosomes of the H3K4me3 mark at the TSSs of thousands of Smp predicted genes [72]. More importantly, we identified the presence of the H3K4me3 mark at the TSSs of 79 out of 232 putative novel protein-coding genes identified by our RNA-Seq, which provided additional evidence of the regulation of these novel putative genes by histone modification. We also found the H3K4me3 TSS histone mark for another 525 re-structured Smp gene models (out of 2,083 Smp gene models with their 5′-ends extended by our RNA-Seq data), thus providing additional confirmation that the original gene model predictions for those 525 genes were improved by using this new 5′-end information. For those genes whose predicted TSS genome positions do not intersect with H3K4me3 peaks, the distance to the closest H3K4me3 peak could indicate the most probable TSS positions [40]; for the Smp predictions that are distant from a H3K4me3 peak, we found that this distance was between 1–2 kbp upstream from the predicted TSS.

Finally, the novel protein-coding genes detected in our transcriptome data are complementary to the existing *S*. *mansoni* gene predictions and annotations, allowing the discovery of genes with possible biological relevance to the parasite. We suggest that among other genes, the SmMEIG gene should be investigated as a potential regulator of parasite sexual reproduction and egg laying. Attention could also be given to the divergent Lifeguard genes SmDLFG1 and 2, two possible inhibitor regulators of cell apoptosis. Interestingly, examining the genomes and gene predictions of other platyhelminths did not provide any evidence of close orthologs, suggesting that this protein would be specific to schistosome species among the platyhelminths. By examining the phylogenetic tree (Fig G in S1 Text), it is possible to observe that the branch that contains the two new SmDLFGs from *S*. *mansoni* contains no other protein from species outside the *Schistosoma* genus, while its branch length is very long. Therefore, it appears to be an isoform specific to this genus that was probably subjected to a positive selection process at some point during its evolution. A possible explanation for this phenomenon would be the co-optation of this protein for some process related to host-parasite interaction, as the new SmDLFGs are transmembrane proteins (see Fig F in S1 Text); in fact, it was previously demonstrated in the *Rickettsiaceae* model that genes coding for parasite membrane proteins tend to display positive selection in relation to genes for membrane proteins of non-parasites [73]. The finding that the new SmDLFGs are highly expressed in females compared with males and eggs (Fig 4) is interesting because the genes from this family are described in humans as encoding an apoptosis inhibitor protein; females are known to be more resistant to drug treatments than males, and the high expression of a possible apoptosis inhibitor protein should be considered when repurposing apoptosis-inducing cancer drugs to treat schistosomiasis [74].

Other putative new genes and gene fragments detected here are likely involved in different biological processes, such as metabolism, development, morphogenesis and cell communication, which raises the possibility of finding and confirming missing genes in the parasite’s molecular pathways.

We also confirmed in this study that thousands of lncRNAs (> 200 nt) are transcribed in *S*. *mansoni* [6,75], and many of these non-coding RNAs may possibly exert regulatory functions that have yet to be explored [75]. In fact, Guttman and co-workers [76] showed that in mammals, there is a class of conserved lncRNAs (i.e., the large intervening non-coding RNAs, lincRNAs) whose transcription and processing appear to be similar to protein-coding genes, with Pol II transcription, 5´-capping and poly-adenylation. Since then, a number of other studies have confirmed that most lncRNAs are poly-adenylated, although there are also non-poly-adenylated ones. Our RNA-Seq cDNA libraries have captured polyA+ RNAs, and the informatics analysis of the sequenced RNAs has identified thousands of long transcripts with non-coding potential, thus providing additional evidence for the existence of such poly-adenylated long noncoding transcripts in *S*. *mansoni*. Cross-reference with known transposon sequences showed that these transcripts do not originate from transcribed repeats. In fact, these lncRNAs are part of the polyA+ RNA pool of a normal eukaryotic cell, and in human cancer cells one of the best characterized consequences of an altered expression of lncRNAs is an important change in deposition of regulatory histone marks at the promoter regions of oncogenes and tumor-suppressor genes [77]. The lncRNA sub-population of a cell, which has long been overlooked, is now being studied by a wide variety of methods [78] that are revealing lncRNAs as an important layer of information that in many instances controls transcription, translation, imprinting, histone modifications, among other functions in the eukaryotic cells.

Additionally, the data discussed in this work are available in a new *S*. *mansoni* Genome Browser Database (http://schistosoma.usp.br/), which uses the UCSC Genome Browser in a Box platform [36] and provides a *S*. *mansoni* public data-searching tool (using the option “Tools > Blat” with a query sequence, or entering a gene name in the *enter position or search term* window), a convenient data-downloading tool (using the option “Tools > Table Browser”) and a visualization tool in a user-friendly format.

## Supporting Information

S1 Table List of primers used in real-time RT-PCR. (XLSX)

S2 Table List of Smp genes expressed in eggs, females or males, along with their significant (P > 95%) differential expression among the three forms studied. (XLSX)

S3 Table Gene ontology analyses for enriched categories by parasite forms. (XLSX)

S4 Table *S*. *mansoni* Smp genes extended at the 5’- and/or 3’-end, with the genomic coordinates of H3K4me3 marks in the vicinity of the genomic coordinates of those genes. (XLSX)

S5 Table *S*. *mansoni* Smp genes with restructured gene models, resulting from either merging or splitting the predicted neighboring genes. (XLSX)

S6 Table Putative novel *S*. *mansoni* genes (232 genes). (XLSX)

S1 Text This file contains the Supplementary Methods, in addition to seven figures. **Fig A. Schematic description of the modified strand-oriented, 5’-end-first 454-Roche cDNA library construction method. Fig B. Housekeeping gene candidates selected to identify a normalizer gene among eggs, females and males. Fig C. Venn diagram of Smp genes expressed in *S*. *mansoni* eggs and female and male adult worms.** Number of Smp genes expressed in each parasite stage, of which over half (3,443 Smp genes) are expressed in common in eggs and female and male adult worms. **Fig D. RNA-Seq read coverage along the gene length of all *S*. *mansoni* genes.** Distribution of all filtered RNA-Seq reads along the gene body (5′- to 3′-end) using read alignment with Tophat2 and the RSeQC package. For each Smp gene, the full length was normalized to 100. **Fig E. H3K4me3 pattern around transcription start sites.** Patterns of H3K4me3 frequency distribution surrounding the TSSs of expressed Smp genes with the histone mark near the 5′-end (within +/- 500 bp) (blue line) compared with the expressed Smp genes without the histone mark close to the 5′-end (red line). A random set of 1,000 genome sequences was used as a control (gray line). **Fig F. Comparison of membrane topology between the new *S*. *mansoni* SmDLFG2 protein and a known Lifeguard protein.** Transmembrane helix profile produced using TMHMM for the sequence of *Homo sapiens* Lifeguard protein 4, representing a typical profile of a Lifeguard protein, and the very similar profile produced for SmDLFG2, one of the new Lifeguard proteins identified in *S*. *mansoni*. **Fig G. Two novel *S*. *mansoni* Lifeguard gene family members and the Bax inhibitor gene family phylogenetic tree.** Phylogenetic tree constructed using Bayesian inference based on multiple alignment of BAX1_I domain (PF01027.16) proteins from diverse eukaryotes.
(DOCX)
