# Supplementary material for: Schistosoma mansoni Egg, Adult Male and Female Comparative Gene Expression Analysis and Identification of Novel Genes by RNA-Seq
Source: PLoS Negl Trop Dis. 2015 Dec 31;9(12):e0004334. doi: 10.1371/journal.pntd.0004334 (PMC4699917; doi:10.1371/journal.pntd.0004334)
Supplement: S1 Text — Fig A. Schematic description of the modified strand-oriented, 5’-end-first 454-Roche cDNA library construction method. Fig B. Housekeeping gene candidates selected to identify a normalizer gene among eggs, females and males. Fig C. Venn diagram of Smp genes expressed in S. mansoni eggs and female and male adult worms. Number of Smp genes expressed in each parasite stage, of which over half (3,443 Smp genes) are expressed in common in eggs and female and male adult worms. Fig D. RNA-Seq read coverage along the gene length of all S. mansoni genes. Distribution of all filtered RNA-Seq reads along the gene body (5′- to 3′-end) using read alignment with Tophat2 and the RSeQC package. For each Smp gene, the full length was normalized to 100. Fig E. H3K4me3 pattern around transcription start sites. Patterns of H3K4me3 frequency distribution surrounding the TSSs of expressed Smp genes with the histone mark near the 5′-end (within +/- 500 bp) (blue line) compared with the expressed Smp genes without the histone mark close to the 5′-end (red line). A random set of 1,000 genome sequences was used as a control (gray line). Fig F. Comparison of membrane topology between the new S. mansoni SmDLFG2 protein and a known Lifeguard protein. Transmembrane helix profile produced using TMHMM for the sequence of Homo sapiens Lifeguard protein 4, representing a typical profile of a Lifeguard protein, and the very similar profile produced for SmDLFG2, one of the new Lifeguard proteins identified in S. mansoni. Fig G. Two novel S. mansoni Lifeguard gene family members and the Bax inhibitor gene family phylogenetic tree. Phylogenetic tree constructed using Bayesian inference based on multiple alignment of BAX1_I domain (PF01027.16) proteins from diverse eukaryotes. (DOCX) [file pntd.0004334.s007.docx]

**Supporting Information for**

***Schistosoma mansoni* Egg, Adult Male and Female**

**Comparative Gene Expression Analysis and**

**Identification of Novel Genes by RNA-Seq**

Letícia Anderson^1^, Murilo S. Amaral^1,2^, Felipe Beckedorff^1^, Lucas F. Silva^1^,

Bianca Dazzani^1^, Katia C. Oliveira^3#a^, Giulliana T. Almeida^1^, Monete R. Gomes^1^,

David S. Pires^1,2^, João C. Setubal^1,4^, Ricardo DeMarco^5^, Sergio Verjovski-Almeida^1,2^*

1 – Departamento de Bioquímica, Instituto de Química, Universidade de São Paulo, 05508-900 São Paulo, SP, Brazil; 2 – Instituto Butantan, 05503-900 São Paulo, SP, Brazil; 3 – Núcleo de Enteroparasitas, Centro de Parasitologia e Micologia, Instituto Adolfo Lutz, 01246-902 São Paulo, SP, Brazil; 4 – Virginia Bioinformatics Institute, Virginia Tech, Blacksburg, VA 24060, USA; 5 – Instituto de Física de São Carlos, Universidade de São Paulo, 13566-590 São Carlos, SP, Brazil

^#a^ Current address: Disciplina de Parasitologia, Departamento de Microbiologia, Imunologia e Parasitologia, Escola Paulista de Medicina, Universidade Federal de São Paulo, 04023-062 São Paulo, SP, Brazil.

**Supplementary Methods page 2**

**Fig A page 2**

**Fig B page 5**

**Fig C page 6**

**Fig D page 7**

**Fig E page 8**

**Fig F page 9**

**Fig G page 10**

**References page 11**

# Supplementary Methods

**Strand-oriented cDNA library preparation**

We had previously developed a method for generating strand-oriented cDNA libraries for 454 sequencing [1], and we have here improved the protocol, aiming to correct for the tendency toward preferential sampling of the 3′-end of the transcripts verified in the previously described method [1]. In this strand-oriented, 5′-end-first cDNA library method, the design of primers was modified so that sequencing starts from the 5′-end of transcripts. Thus, the positions of the primer-A and primer-B adaptors relative to the RNA were inverted in this method, compared with the previous method [1]. Figure A provides a schematic overview of the new method.


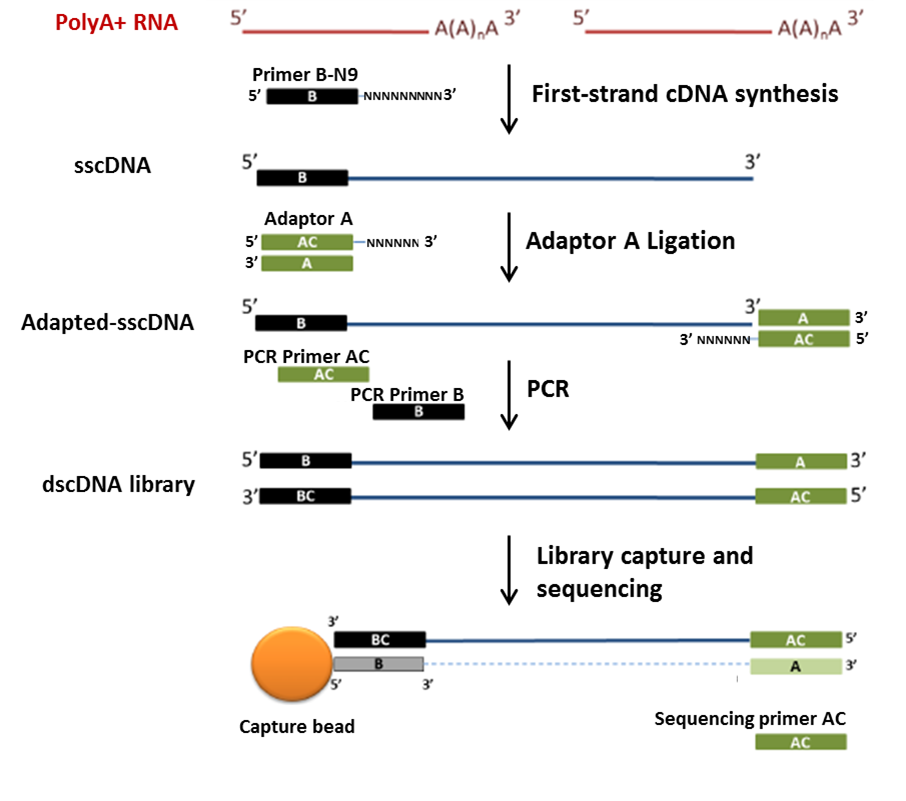


**Fig A.** **Schematic overview of the modified strand-oriented, 5’-end-first 454-Roche cDNA library construction method.**

To prepare dscDNA libraries for Roche/454 sequencing, a primer was synthesized with the Roche primer–B adaptor sequence [1] added to the 5′-end of a 9-mer fully degenerate primer (Primer B-N_9_, top black box in Fig A). The sequence of this adaptor-B-random primer is C*C*T* A*TC CCC TGT GTG CCT TGG CAG TCT CAG N_9_ (IDT, Integrated DNA Technologies). (Note: * are phosphorothioated bases). This primer was used for first-strand cDNA synthesis, and in this way, the primer-B adaptor sequence is now located at the first-strand cDNA in a position that corresponds to the RNA 3’-end. First-strand cDNAs were synthesized using the SuperScript III First-Strand Synthesis Super Mix reverse transcriptase (Invitrogen) in a total reaction volume of 20 μl, with 1 μg polyA^+^ RNA and 0.312 μM of adaptor-B-random primer, followed by 20 min of RNase H (2U) and RNase A (0.05 mg/mL) treatment at 37 °C and then 5 min at 85 °C. The reaction was then cleaned up using RNAClean beads (Agencourt), and the dscDNA-Adaptor-A (6.67 μM) was ligated to the purified single-stranded cDNAs using 2000 units of T4 DNA Ligase enzyme (New England BioLabs) at 16 °C for 2 h. Primer A containing double-stranded cDNA adaptor A (Adaptor-A, green boxes in Fig A) was prepared using cDNA Adaptor Oligo A Prime (5’-C*C*A* T*CT CAT CCC TGC GTG TCT CCG ACT CAG NN*N* N*N*N Phos-3’), depicted as AC in figure A, and cDNA Adaptor Oligo A (5’-Phos C*T*G* A*GT CGG AGA CAC GCA GGG ATG AG*A* T*G*G-3’), depicted as A in figure A. Note: * are phosphorothioated bases; 5’-Phos and Phos-3’ are phosphorylated bases. Equal molar amounts of cDNA Adaptor Oligo A Prime and cDNA Adaptor Oligo A were mixed, and the following program was used to generate the dscDNA-Adaptor-A: 5 min at 80 °C, 7 min at 65 °C, followed by 8 steps of 7 min each where the temperature was lowered by 5 °C per step down to 25 °C, hold at 4 °C.

After the ligation of dscDNA-Adaptor-A, the resulting single-stranded cDNA (Adapted-sscDNA in Fig A) libraries were purified twice with RNAClean (Agencourt) according to the manufacturer’s instructions except for the amount of beads, which was reduced to 1.6x the sample volume. The purified sscDNA libraries were analyzed on an RNA 6000 Pico chip on a 2100 Bioanalyzer (Agilent Technologies) to confirm a size distribution between 450 to 1000 nucleotides, then quantified using the Quant-iT Ribogreen RNA Assay Kit (Invitrogen) on a SpectraMax M2 (Molecular Devices) instrument, following the manufacturer’s instructions. The sscDNA libraries were PCR amplified with 2 μM each of PCR Primers A (5’-C*C*A* T*CT CAT CCC TGC GTG TCT C*C*G* A*C-3’) and B (5’-C*C*T* A*TC CCC TGT GTG CCT TGG C*A*G* T*C-3’), 400 μM dNTPs, 1X Advantage 2 buffer and 1 μL of Advantage 2 polymerase mix (Clontech). The amplification reaction was performed at 96 °C for 4 min; 94 °C for 30 s, 64 °C for 30 s, repeating steps 2 and 3 for a total of 10 cycles for male and female libraries and 17 cycles for egg libraries, followed by 68 °C for 3 min. The samples were purified using two rounds of AMPure beads (Agencourt) and quantified using the Quant-iT PicoGreen DNA Assay Kit (Invitrogen). The resulting dscDNA libraries were analyzed on a High Sensitivity DNA Kit on a 2100 Bioanalyzer (Agilent Technologies) and used for sequencing.

The configuration of primer localization at the 3’- and 5’-ends of the messages used here is the inverse of our previous method [1] and provided an enriched sequencing of the 5′-end of messages, as shown in the Results section. Moreover, this inversion eliminated the portion of reads that had the polyA tract as a starting point for sequencing, which had previously resulted in poor sequencing quality due to known problems of the 454 sequencer with homopolymers. In addition, the RNA fragmentation step [1] was eliminated from the new protocol, which resulted in reverse transcription now being performed using whole transcripts as templates.

**Housekeeping gene determination**

**
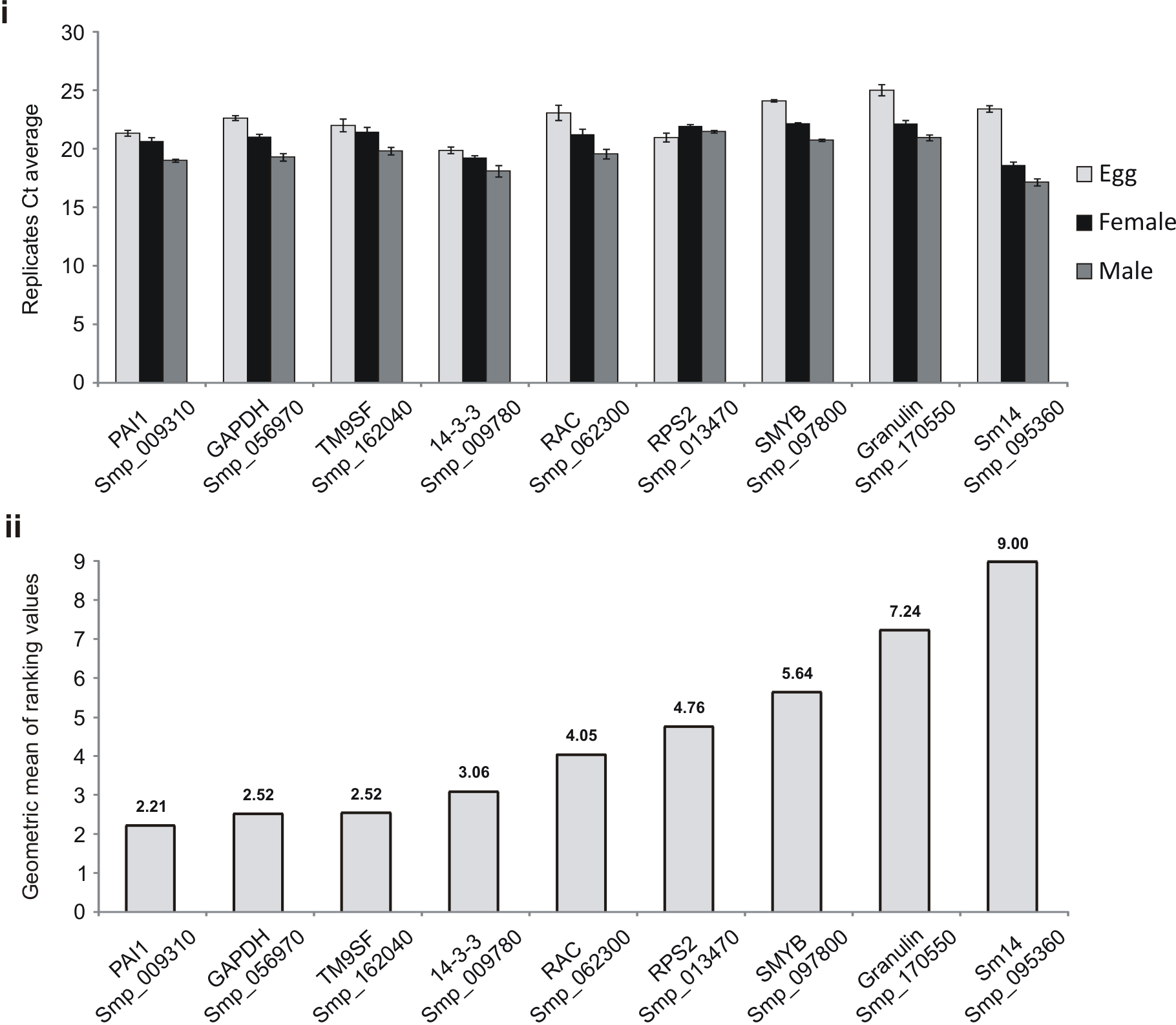
**

**Fig B. Housekeeping gene candidates selected to identify a normalizing gene among eggs, females and males.** (i) Nine selected genes that showed no evidence of differential expression among parasite forms in the RNA-Seq data were evaluated by qPCR with three biological replicates for each form. The gene cycle threshold (Ct) for each form is shown. (ii) The qPCR data were analyzed by the RefFinder tool [2], which calculates the geometric mean of ranking values, obtained by four different algorithms, for each gene among the three forms. The gene with the lowest ranking geometric mean is the most stable gene among the three forms and was used for the normalization of additional qPCR data.

**
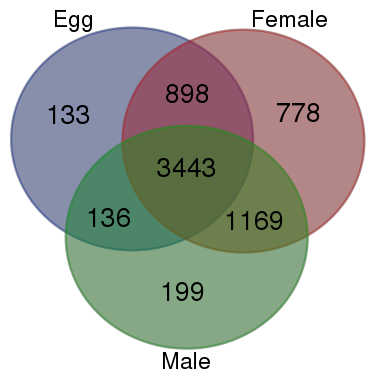
**

Fig C. **Venn diagram of Smp genes expressed in *S. mansoni* eggs and female and male adult worms.**

Number of Smp genes expressed in each parasite form, of which over half (3,443 Smp genes) are expressed in common in eggs and female and male adult worms.


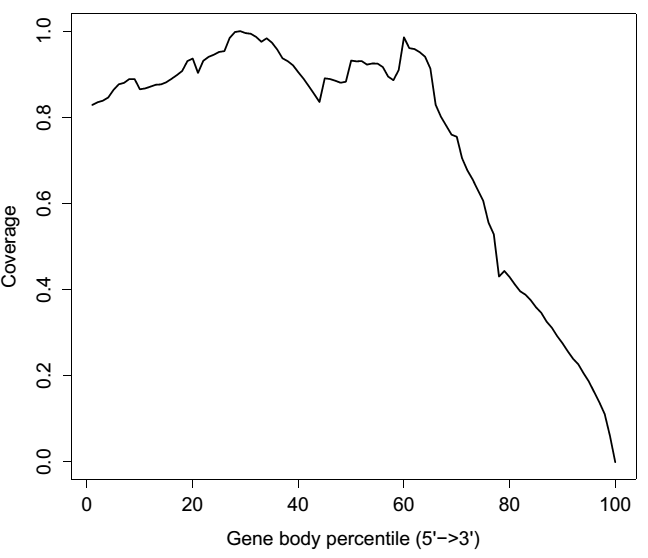


Fig D. **RNA-Seq read coverage along the gene length of all *S. mansoni* genes.**

Distribution of all filtered RNA-Seq reads along the gene body (5′- to 3′-end) using read alignment with Tophat2 [3] and the RSeQC package [4]. For each Smp gene, the full length was normalized to 100.


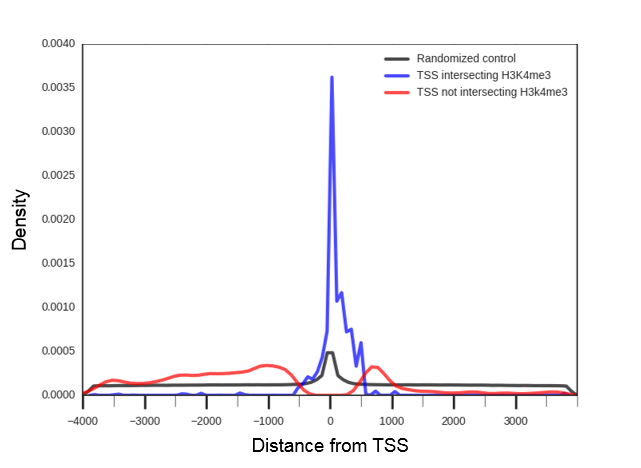


Fig E. **H3K4me3 pattern around transcription start sites.**

The patterns of H3K4me3 frequency distribution surrounding the TSSs of expressed Smp genes with the histone mark near the 5′-end (within +/- 500 bp) (blue line) compared with the expressed Smp genes without the histone mark close to the 5′-end (red line). A random set of 1,000 genome sequences was used as a control (gray line).


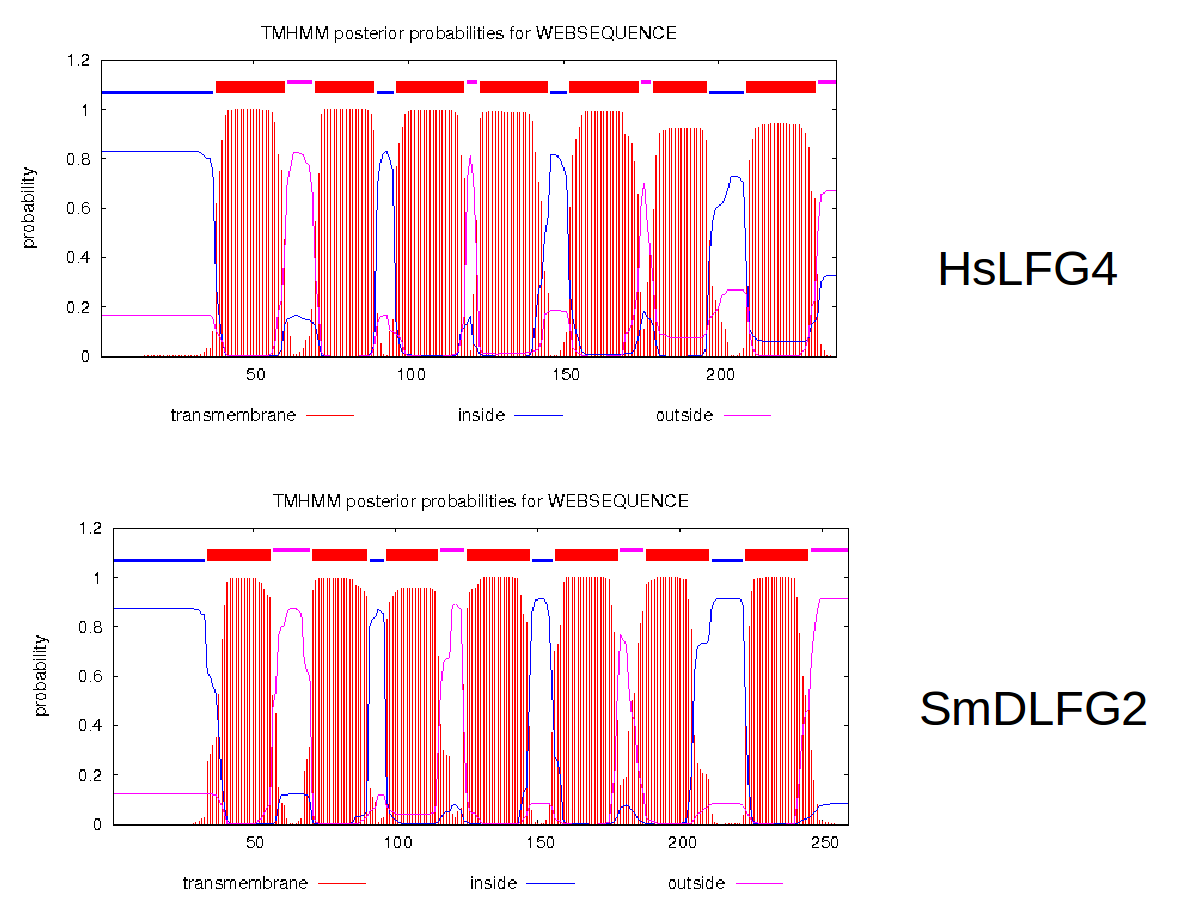


Fig F. **Comparison of membrane topology between the new *S. mansoni* SmDLFG2** **protein and a known Lifeguard protein.**

Transmembrane helix profile produced using TMHMM for the sequence of *Homo sapiens* Lifeguard protein 4, representing a typical profile of a Lifeguard protein, and the very similar profile produced for SmDLFG2, one of the new Lifeguard proteins identified in *S. mansoni*.

**
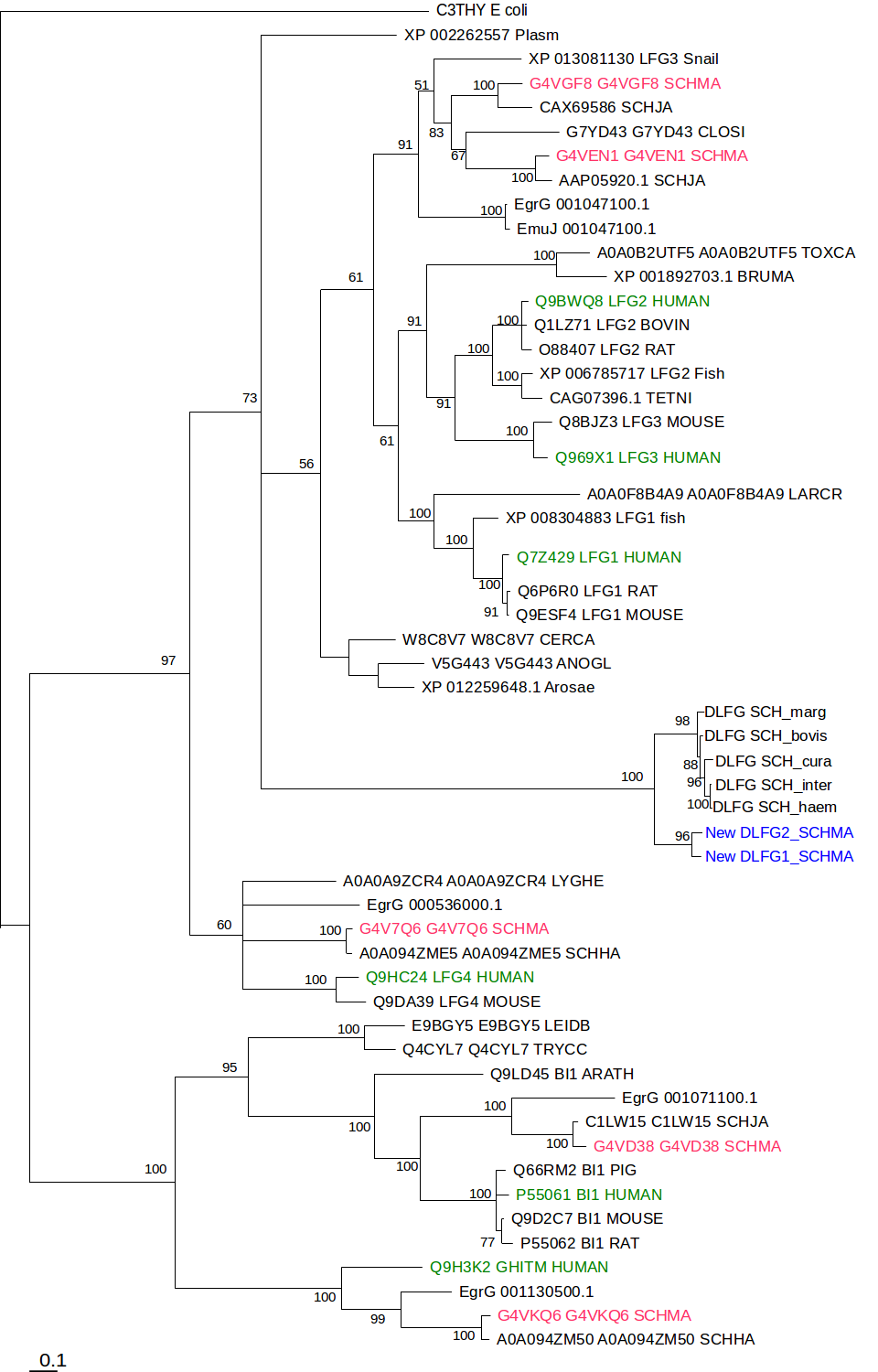
**

Fig G. **Two novel *S. mansoni* Lifeguard gene family members and the Bax inhibitor gene family phylogenetic tree**.

Phylogenetic tree constructed using Bayesian inference based on multiple alignment of BAX1_I domain (PF01027.16) proteins from diverse invertebrate and vertebrate eukaryotes. Numbers next to nodes indicate associated posterior probabilities. Five previously described *S. mansoni* proteins displaying a complete Bax Inhibitor 1 domain (BI-1) are shown with their UniProt accession numbers in red, and human proteins are marked in green. The two newly identified *S. mansoni* Lifeguard proteins are marked in blue and were used in a phylogenetic analysis that included other representative proteins of this family recovered from the UniProt database (accession numbers indicated for each protein entry). A sequence of a BAX1_I domain from a prokaryote (*E. coli*) was utilized as an outgroup.

**References**

1. Almeida GT, Amaral MS, Beckedorff FC, Kitajima JP, DeMarco R, et al. (2012) Exploring the *Schistosoma mansoni* adult male transcriptome using RNA-seq. Exp Parasitol 132: 22-31.

2. Fu W, Xie W, Zhang Z, Wang S, Wu Q, et al. (2013) Exploring valid reference genes for quantitative real-time PCR analysis in Plutella xylostella (Lepidoptera: Plutellidae). Int J Biol Sci 9: 792-802.

3. Trapnell C, Roberts A, Goff L, Pertea G, Kim D, et al. (2012) Differential gene and transcript expression analysis of RNA-seq experiments with TopHat and Cufflinks. Nat Protoc 7: 562-578.

4. Wang L, Wang S, Li W (2012) RSeQC: quality control of RNA-seq experiments. Bioinformatics 28: 2184-2185.
